# Pathogen genomics study of an early medieval community in Germany reveals extensive co-infections

**DOI:** 10.1186/s13059-022-02806-8

**Published:** 2022-12-13

**Authors:** Joanna H. Bonczarowska, Julian Susat, Barbara Mühlemann, Isabelle Jasch-Boley, Sebastian Brather, Benjamin Höke, Susanne Brather-Walter, Valerie Schoenenberg, Jonathan Scheschkewitz, Gabriele Graenert, Dirk Krausse, Michael Francken, Terry C. Jones, Joachim Wahl, Almut Nebel, Ben Krause-Kyora

**Affiliations:** 1 Institute of Clinical Molecular Biology, Kiel University, 24105 Kiel, Germany; 2 Institute of Virology, Charité – Universitätsmedizin Berlin, corporate member of Freie Universität Berlin, Humboldt-Universität zu Berlin, and Berlin Institute of Health, 10117 Berlin, Germany; 3 German Centre for Infection Research (DZIF), partner site Charité, 10117 Berlin, Germany; 4 Institute for Archaeological Sciences, Palaeoanthropology Working Group, University of Tübingen, Rümelinstrasse 23, 72070 Tübingen, Germany; 5 Institute of Archaeology, Freiburg University, Belfortstraße 22, 79085 Freiburg, Germany; 6 Landesamt für Denkmalpflege im Regierungspräsidium Stuttgart, Berliner Straße 12, 73728 Esslingen, Germany; 7 Museum im Ritterhaus, Ritterstraße 10, 77652 Offenburg, Germany; 8 Landesamt für Denkmalpflege im Regierungspräsidium Stuttgart, Konstanz, Germany; 9 Center for Pathogen Evolution, Department of Zoology, University of Cambridge, Cambridge, CB2 3EJ UK

**Keywords:** Ancient DNA, Pathogen genomics, Ancient genomics, Variola virus, Smallpox, Leprosy, Hepatitis B virus, Parvovirus B19, Pathogen evolution, Infectious diseases

## Abstract

**Background:**

The pathogen landscape in the Early European Middle Ages remains largely unexplored. Here, we perform a systematic pathogen screening of the rural community Lauchheim “Mittelhofen,” in present-day Germany, dated to the Merovingian period, between fifth and eighth century CE. Skeletal remains of individuals were subjected to an ancient DNA metagenomic analysis. Genomes of the detected pathogens were reconstructed and analyzed phylogenetically.

**Results:**

Over 30% of the individuals exhibit molecular signs of infection with hepatitis B virus (HBV), parvovirus B19, variola virus (VARV), and *Mycobacterium leprae*. Seven double and one triple infection were detected. We reconstructed four HBV genomes and one genome each of B19, VARV, and *M. leprae*. All HBV genomes are of genotype D4 which is rare in Europe today. The VARV strain exhibits a unique pattern of gene loss indicating that viruses with different gene compositions were circulating in the Early Middle Ages. The *M. leprae* strain clustered in branch 3 together with the oldest to-date genome from the UK.

**Conclusions:**

The high burden of infectious disease, together with osteological markers of physiological stress, reflect a poor health status of the community. This could have been an indirect result of the climate decline in Europe at the time, caused by the Late Antique Little Ice Age (LALIA). Our findings suggest that LALIA may have created an ecological context in which persistent outbreaks set the stage for major epidemics of severe diseases such as leprosy and smallpox hundreds of years later.

**Supplementary Information:**

The online version contains supplementary material available at 10.1186/s13059-022-02806-8.

## Background

The pathogen landscape of the Early Middle Ages in Europe (late fifth–tenth century CE) remains largely unexplored. Bacterial and viral genomes from this period have so far only been detected in a few cases across the continent, such as a hepatitis B virus (HBV) [1, 2], variola virus (VARV) [3], and *Mycobacterium leprae* (*M. leprae*) [4]. These findings, although important in the context of pathogen evolution, are widely dispersed in time and space and do not significantly contribute to the understanding of the prevalence of infectious diseases in a specific locale during that period.

Prevalence rates of infectious diseases in pre-modern populations are uncertain, as they are usually based only on historical records [5] and paleopathological analysis of human skeletal and mummified remains [6]. Ancient pathogen genomics is an additional approach that can confirm the infection, even when there are no lesions present on skeletal remains or when disease manifestation is atypical. Combined analysis of the written record, skeletal remains, and genomic information can provide valuable knowledge of historical infectious disease presence.

Here, we perform a systematic metagenomic pathogen screening of 70 individuals representing 89% of all people buried within the Lauchheim “Mittelhofen” village (present-day Germany) in the Alemannic duchy of the Merovingian kingdom. The Merovingian period (late fifth century to 751 CE) was an important transitional phase, paving the way for what became known as the culture of Medieval Europe [7]. Here, we present molecular evidence of infection with four different pathogens (human parvovirus B19 (B19), HBV, variola virus, and *M. leprae*) in 22 individuals. This study provides insights into the diversity and prevalence of infectious diseases in a rural community during the late seventh and eighth century CE.

## Results

**Fig. 1:**
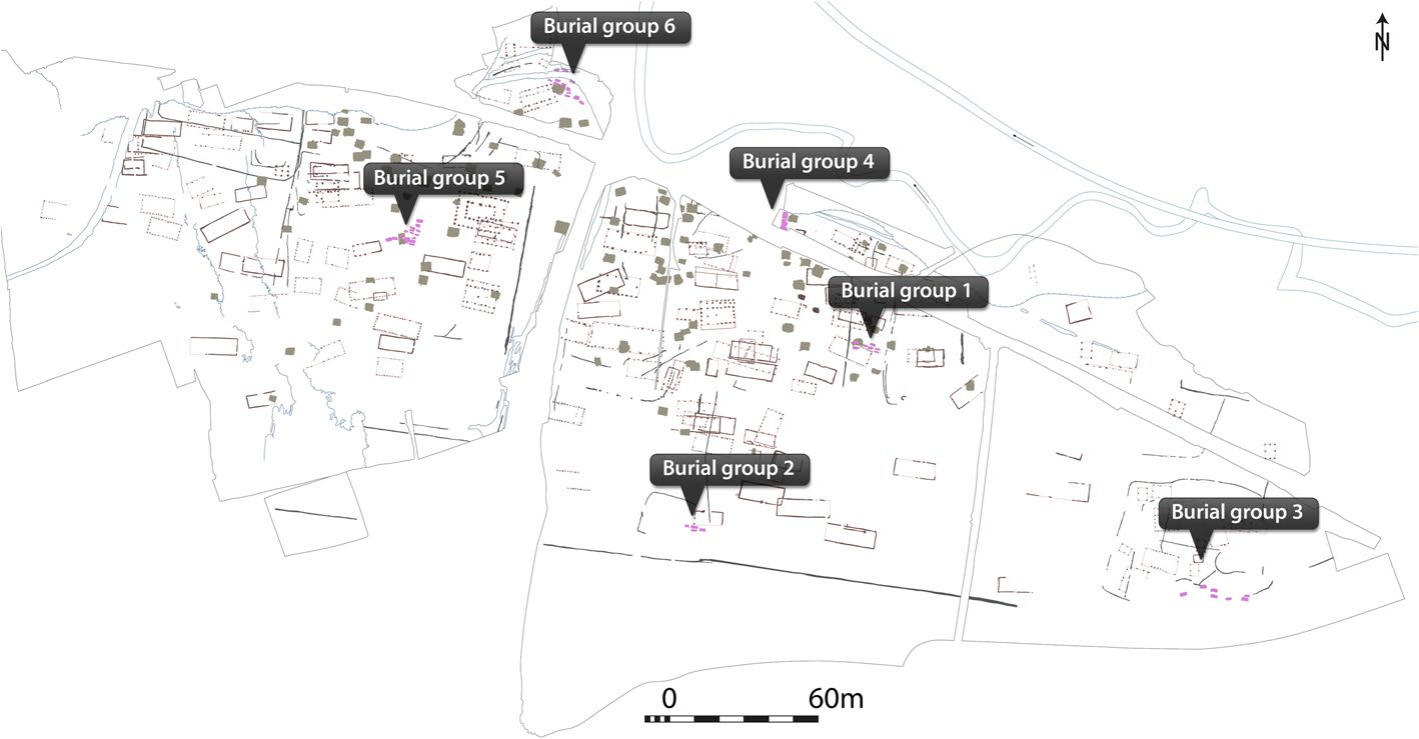
Map of the archeological features of the Lauchheim “Mittelhofen” settlement. Burial groups are indicated. Burial groups are thought to be associated with distinguishable farmsteads. Purple rectangles represent the graves, while gray squares indicate pit houses and dotted-line rectangles post pits of houses

Seventy-nine burials, in six burial groups (Fig. 1), were excavated at the Lauchheim “Mittelhofen” settlement. Based on archeological and radiocarbon evidence, the burials date to the late seventh and eighth century CE. It is thought that inhabitants buried the deceased at the edge of their property [8]. In total, metagenomic samples from 70 individuals were sequenced in this study (Additional file 1: Tab. S1).

### Pathogen screening

Bacterial and viral screening of metagenomic samples revealed that DNA of four pathogens was present in 22 individuals: B19 (*n* = 21), HBV (*n* = 8), VARV (*n* = 1), and *M. leprae* (*n* = 1) (Table 1 and Additional file 1: Tab. S2 and S3). In six cases, a double infection with B19 and HBV was observed. A double infection with B19 and variola virus was found in one individual (G31) and three different pathogens (B19, HBV, and *M. leprae*) were detected in a sample of an adolescent male (G83). Data enrichment allowed the reconstruction and analysis of four HBV (G78, G79, G27, and G83), one B19 (G83), the VARV (G31) and the *M. leprae* genomes (G83) (Table 1). Based on these data, we report on the crude period prevalence (CPP) of infectious diseases in the Lauchheim settlement [6]. CPP is defined as the percentage of individuals in a population (%) that exhibit a molecular sign of infection over the period of time in which the settlement was used as a burial ground (approx. a century). Thus, the CPP indicates the prevalence of infections over three to four human generations, during which the pathogen landscape can change. The CPP calculated here for the Lauchheim population was 31.4%. When adult and subadult individuals are considered separately, the CPP was 29.1% for adults and 40% for subadults. Authenticity of the ancient origin of the pathogens was confirmed by the presence of typical ancient DNA (aDNA) damage patterns (Additional file 1: Tab. S4) and the ancestral positions of the pathogen genomes in phylogenetic trees (see below).

**Table 1:** Individuals from the Lauchheim “Mittelhofen” settlement with detected infection

No.	Grave number/individual ID	Sex	Age [years]	Pathogen	Burial group	^**14**^C (2 σ) [years cal CE]	Physiological stress indicators [9]
1	5	M	40–50	B19	1	-	*Cribra orbitalia*
2	7	M	Around 25	B19	1	-	*Cribra orbitalia*, *cribra femoris*, enamel hypoplasia
3	22†	M	Around 15	B19	3	-	*Cribra orbitalia*
4	23	M	21–23	B19	3	-	*Cribra cranii*, *cribra femoris*
5	24	F	Senile	B19	3	-	*Cribra orbitalia*, *cribra cranii*, *cribra femoris*,
6	37	M	Around 25	B19	4	674–820	*Cribra orbitalia*, *cribra cranii* and *cribra femoris*, enamel hypoplasia
7	56	M	Early mature	B19	5	-	*Cribra orbitalia*, *cribra cranii*
8	63	M	25–30	B19	6	-	*Cribra cranii*, enamel hypoplasia
9	67^†^	F	16–20	B19	6	-	Enamel hypoplasia
10	69	F	40–50	B19	6	-	-
11	70	F	30–40	B19	6	-	Enamel hypoplasia
12	82	F	Mature	B19	6	-	*Cribra orbitalia*, enamel hypoplasia
13	86	M	Around 60	B19	6	644–770	Enamel hypoplasia
14	78	M	40–50	HBV^g^	6	647–771	*Cribra femoris*
15	31	F	25–30	B19, VARV^g^	4	685–879	*Cribra cranii*
16	16	F	20–25	B19, HBV	2	-	*Cribra orbitalia*, *cribra femoris*
17	27	M	30–35	B19, HBV^g^	3	703/704^#^	*Cribra orbitalia*, *cribra cranii*, enamel hypoplasia
18	29	F	20–25	B19, HBV	4	665–773	*Cribra cranii*, *cribra femoris*, enamel hypoplasia
19	46†	PF^$^	12–14	B19, HBV	5	-	*Cribra orbitalia*, *cribra cranii*, *cribra femoris*
20	66^†^	M	6–8	B19, HBV	6	-	Enamel hypoplasia
21	79^†^	M	13–14	B19, HBV^g^	6	650–771	*Cribra orbitalia*, *cribra femoris*, enamel hypoplasia
22	83^†^	M	13–15	B19^g^, HBV^g^, *M. leprae*^g^	6	656–772	*Cribra orbitalia*, *cribra cranii*

^g^Genome reconstructed, *M* male, *F* female, *PF* probable female, ^$^based on osteological analysis [9], ^†^subadult, ^#^based on dendrochronology

### Genome-wide analysis of the identified pathogens

#### Hepatitis B virus

**Fig. 2:**
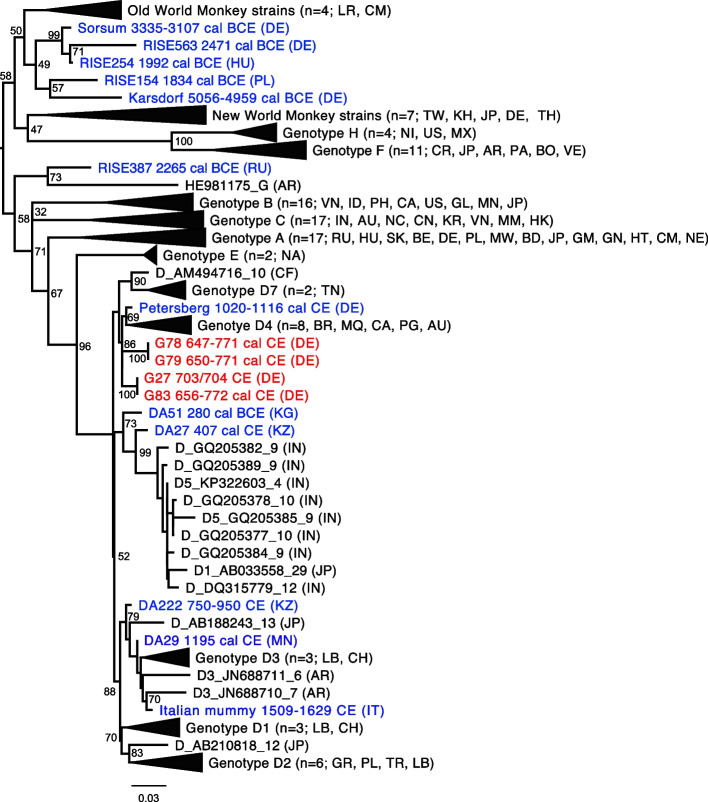
A maximum-likelihood tree illustrating the phylogenetic position of the HBV strains from Lauchheim (red). Other ancient strains are marked in blue and modern HBV are shown in black. Numbers at each node are bootstrap support over 500 replications. The tree includes 130 strains (109 modern and 21 ancient). Country codes can be found in the Additional file 1: Table S6. Dates are provided for ancient strains

From the eight HBV-positive samples (CPP = 11.4%), we generated four almost complete viral genomes (G78, G79, G27, and G83) ranging in coverage from 96 to 99.9% (Additional file 1: Tab. S5). In the phylogeny, all four strains were basal to genotype D4 (Fig. 2 and Additional file 1: Fig. S1), clustering in two pairs of identical strains (G78 and G79, G27 and G83). Each genome carried the 33nt deletion in *preS1* typical of genotype D and a complete sequence of the HBe-antigen.

#### Human parvovirus B19

**Fig. 3:**
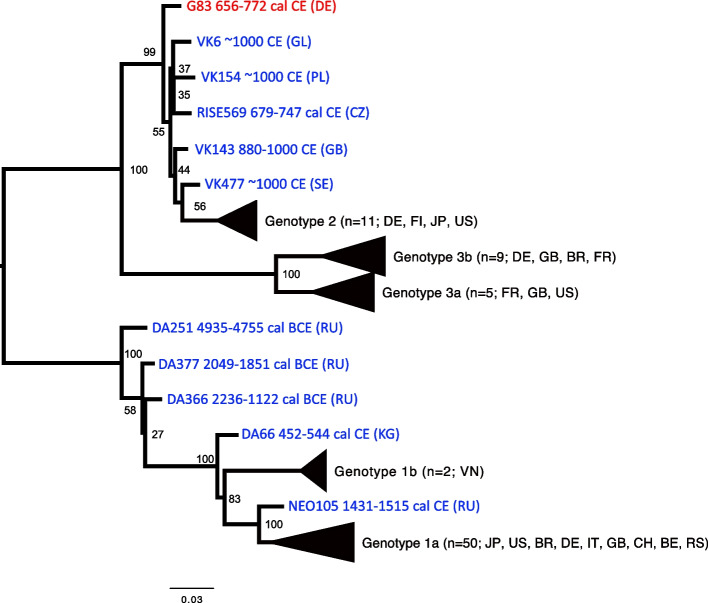
A maximum-likelihood tree illustrating the phylogenetic position of the B19 strain from Lauchheim (red). Other ancient strains are marked in blue and modern B19 are shown in black. Numbers at each node are bootstrap support over 500 replications. The tree includes 88 strains (77 modern and 11 ancient). Country codes can be found in the Additional file 1: Table S6. Dates are provided for ancient strains

From the 21 parvovirus-positive samples (CPP = 30%), one complete B19 genome (G83) was reconstructed, with highest nucleotide identity to the modern genotype 2 (Additional file 1: Tab. S5). In the phylogenetic tree, G83 was basal to the cluster of several ancient European and modern genotype-2 strains (Fig. 3 and Additional file 1: Fig. S2).

#### Variola virus

**Fig. 4:**
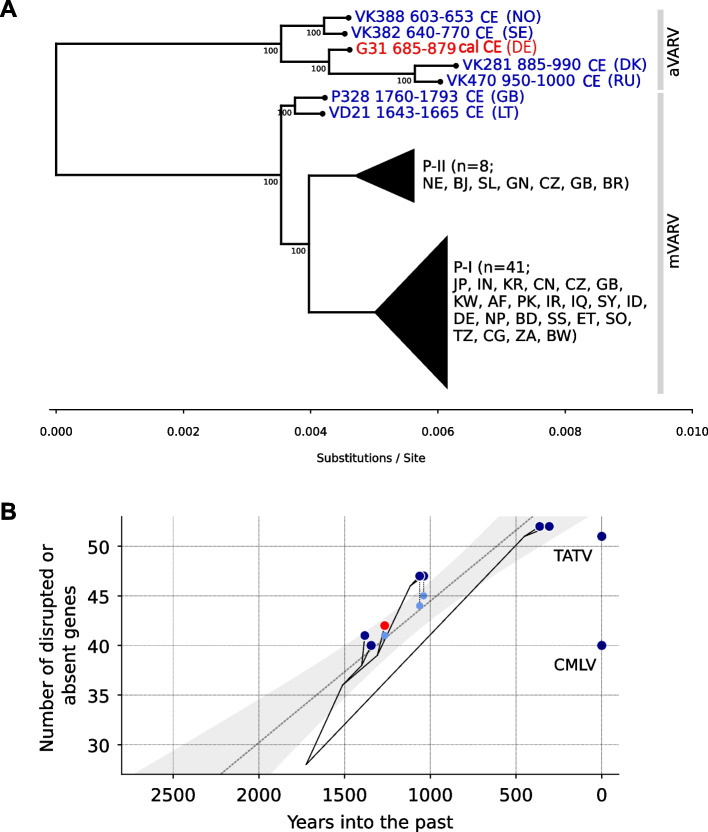
G31 variola virus from Lauchheim exhibits intermediate genetic characteristics in relation to other ancient strains. **A** A maximum-likelihood tree illustrating the phylogenetic position of the VARV strain from Lauchheim (red) among other ancient (blue) and modern (black) strains. Numbers at each node are bootstrap support. The tree was inferred using IQTree, with 10,000 ultrafast bootstrap replicates. The tree includes 56 sequences (49 modern and 7 ancient). P-I and P-II refer to VARV primary clade I (corresponding to VARV major and East, Central and South African VARV sequences) and primary clade II (comprising of VARV alastrim minor and West African sequences), respectively. Country codes can be found in the Additional file 1: Table S6. Dates are provided for ancient strains. **B** Temporal trend of gene inactivation, with the number of inactivated genes shown on the *y*-axis and the *x*-axis indicating time into the past. Phylogenetic relationships between viruses are indicated by gray lines. Light blue circles indicate the number of inactivated genes when uncertain inactivations are not taken into account. The gray dotted line and shaded area indicate a linear regression of number of genes against time into the past, indicating that a hypothetical virus with a full set of genes existed ~4116 years into the past (*R*: 0.92, *R*^2^: 0.85, slope: 0.014, *y*-intercept: 58.76, stderr: 0.002, *P*: 0.00002)

Sample G31 (685–879 cal CE) had 81 reads mapping to VARV in the initial screening (NC_001611.1). Subsequent capture for viral sequences resulted in the recovery of a near complete genome (98%, 60.8 x coverage) (Additional file 1: Tab. S7). Phylogenetically, the G31 genome clusters within the ancient VARV (aVARV) clade, basal to the strains VK281 and VK480 found in Denmark and Russia, respectively (Fig. 4A) [3]. The dates of the most recent common ancestor of all aVARVs (~1500 years ago), as well as all VARVs (~1700 years ago), are consistent with previous publications [3]. G31 exhibits a pattern of gene inactivation with features from both the VK388-VK382 and VK281-VK470 clades (Fig. 4B and Additional file 1: Fig. S3), congruent with its position in the phylogeny (Fig. 4A). Concerning the genes showing differences in activation patterns across the two branches, G31 shares gene-inactivating mutations with the VK382-VK388 branch in three genes (A39R, C2L, F3L) and with the VK281-VK470 branch in four genes (22682/CPXV020, K1L, 168145/CPXV181, A57R).

#### Mycobacterium leprae

**Fig. 5:**
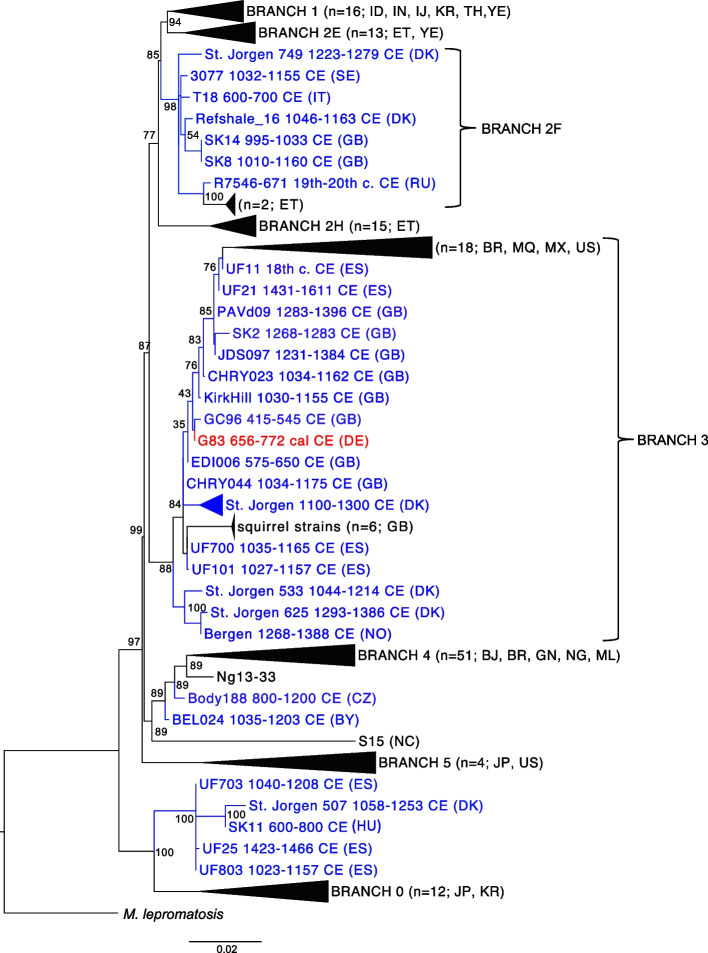
A maximum-likelihood tree illustrating the phylogenetic position of the *M. leprae* strain from Lauchheim (red). Other ancient strains are marked in blue and modern *M. leprae* are shown in black. Numbers at each node are bootstrap support over 500 replications. The tree includes 177 strains (139 modern and 38 ancient). Country codes can be found in the Additional file 1: Table S6. Dates are provided for ancient strains

A genome of *M. leprae* (4 x) was assembled (Additional file 1: Tab. S8) from the skeletal remains of an adolescent male (G83) (Table 1) which showed several pathological lesions (Additional file 1: Tab. S9, Fig. S4 and Fig. S5). In the phylogenetic analysis, G83 was positioned in branch 3, together with modern strains from the Americas (human and nine-banded armadillo) and the UK (red squirrel) as well as human medieval strains from Denmark and the UK (Fig. 5 and Additional file 1: Fig. S6). G83 clustered with the British GC96 sequence dating to the fifth–sixth century CE, forming a separate subbranch.

## Discussion

Pathogen screening of the community buried at the early medieval settlement Lauchheim “Mittelhofen” revealed that more than 30% of the population was positive for least one pathogen at the time of death. We identified seven individuals with concurrent infections with two viruses (G16, G27, G29, G31, G46, G66, and G79) and one individual with a triple infection of HBV, B19, and *M. leprae* (G83) (Table 1).

 Seven percent of adults and 26.6% of subadults had an HBV infection (Table 1), a prevalence which today is considered endemic [10]. Furthermore, the proportion of HBV-infected subadults in Lauchheim was over three times as high as that of adults, which is in accordance with the age-related infection pattern in endemic areas [10]. Although today most HBV infections are asymptomatic or cause mild symptoms, individuals with a compromised immune system are at risk of developing severe complications such as liver failure, cirrhosis, and liver cancer. All HBV genomes were of sub-genotype D4—as previously found in eleventh–twelfth century Petersberg (Germany) [11]. To date, sub-genotype D4 is mainly found in Australasia and Northern America. Thus, the available data point to a temporal change in the geographical distribution of HBV genotype D sub-genotypes in Europe—with early medieval D4 subtypes being replaced with those that are prevalent today, such as D1, D2, and D3 [12]. Interestingly, in sixteenth century Italy, an HBV strain of genotype D3 was already present [13] (Fig. 2). Two pairs (G78 and G79; G27 and G83) of identical genomes were reconstructed (based on sequence comparison). Although the diversity of HBV at the time is unknown, this finding could possibly suggest a direct transmission between these individuals or a common source of infection.

In comparison to HBV, B19 was more abundant, with 30% of the Lauchheim community identified as positive. The CPP estimated for subadults (40%) falls within the prevalence seen today (15-60%). The adult CPP of 27.3% is slightly below the rate expected in the present day (30-60%), suggesting that it might likely be an underestimate. B19 infections can lead to a mild disease in children (fifth disease) and serious fetal conditions in pregnant women. In immunocompromised individuals, the infection may lead to anemia and life-threatening aplastic crisis [14]. Thirteen out of the 21 B19-positive individuals in Lauchheim died before reaching an estimated 30 years of age (62%, in comparison to 41% in the overall population). Although not particularly likely, premature death could have possibly occurred due to complications developed after a B19 infection. In the phylogenetic analysis, the G83 strain (genotype 2) is basal to all modern and ancient genotype 2 genomes recovered to date. Genotype 2 is today mainly found in elderly northern Europeans [15].

Variola virus (VARV) was detected in one individual (G31). VARV is the causative agent of the now-eradicated smallpox. The disease is characterized by high mortality (reaching up to 30%), with symptoms including fever, vomiting, and skin rash [16]. In the phylogenetic analysis, the G31 strain clustered within the known diversity of early medieval VARVs from northern and eastern Europe [3]. These VARVs are distinguished from modern VARVs by a larger number of intact genes. G31 exhibits a pattern of gene loss that is a combination of unique features found in both the VK382-VK388 and VK281-VK470 branch of the medieval VARV clade, showing that viruses with different gene compositions were circulating in the Early Middle Ages. The reductive evolution in modern VARVs is thought to be a result of changed selection pressures due to adaptation to humans as exclusive hosts [17]. Given the transmissibility of modern VARV, it is interesting that we only detected a VARV infection in one Lauchheim inhabitant. The absence of additional VARV-positive samples in Lauchheim may be due to a lower fatality rate [18], or the fact that viremia is not thought to last for the duration of infection [19], and thus the viral DNA was not preserved. Furthermore, genomic differences between the modern and medieval VARVs (Fig. 4 and Additional File 1: Fig. S3) could possibly reflect a reduced efficiency of human-human transmission in ancient VARVs. The epidemiology of VARV in the Early Middle Ages, however, remains unknown.

In addition to viral infections, the pathogen screening revealed the presence of *M. leprae* (causative agent of leprosy) in an adolescent male (G83). Leprosy mainly affects the skin, mucous membranes, and peripheral nerves. Subsequent sensory loss in the hands and feet makes these areas susceptible to injuries and secondary infections, usually leading to deformation and in some cases even loss of extremities. Leprosy per se is not deadly and only approximately 5% of infected individuals exhibit clinical symptoms. As leprosy can cause severe disability as well as visible disfigurement of the limbs and face [20], social stigma of the disease likely surpassed its clinical outcome in terms of suffered consequences. The skeleton of individual G83 exhibits signs suggestive of lepromatous leprosy that take years to develop. Although the adolescent likely had visible facial disfigurement caused by leprosy, he was buried together with the rest of the community, indicating that he might not have been ostracized. Although leprosy was already present in Europe in the Early Middle Ages [21, 22], its prevalence peaked between the twelfth and fourteenth centuries CE. The G83 strain is the oldest found in Germany to date. Two British strains dating to a similar time period were included in the phylogenetic analysis (GC96, fifth–sixth century and EDI006, sixth–seventh century CE). The Lauchheim strain groups in branch 3 together with both British strains, suggesting low diversity of *M. leprae* in the Early Medieval Period [23]. Based on the observed variation (Fig. 5) as well as the temporal and geographic distribution of the medieval strains (Additional file 1: Fig. S7), we can hypothesize an early appearance of branch 3 strains in Europe. The spread may have been facilitated by the expanding Roman Empire [22, 23].

Although the dating of the Lauchheim settlement abuts the Justinian plague (541–543 CE), no traces of the causative *Yersinia pestis* (*Y. pestis*) were found in any of the analyzed samples. Moreover, in the case of the Lauchheim settlement, most likely not all inhabitants were buried within its borders and part of the population might have been buried in the traditional graveyard situated nearby or at another unknown location. Overall, the burials may not be representative of the general Lauchheim population. Excavation and preservation bias also influence the amount of data available for analysis. Inherent limitations of the collected molecular evidence must also be considered. Lack of infection cannot be inferred from pathogen-negative DNA samples as degradation of the genetic material could have erased traces of the microorganism. Thus, the observed infectious disease prevalence in Lauchheim is probably an underestimate.

In the early medieval community of Lauchheim, we observed a high prevalence of infectious diseases caused by four different pathogens. Pathogens were detected in all six burial groups, indicating a broad distribution of infections across the population. The extent of the disease burden becomes even more apparent when considering the fact that pathogens which do not enter the bloodstream are most likely undetectable, due to lack of DNA preservation. Moreover, other types of diseases, such as hereditary, metabolic, and nutritional diseases or cancer, were not accounted for in this investigation. Interestingly, 95.5% of the pathogen-positive individuals (Table 1) and 84.4% of the individuals with no molecular sign of infection exhibited skeletal lesions suggestive of physiological stress. This extremely high burden of lesions indicates poor health in Lauchheim. In one scenario, malnutrition could have increased the level of physiological stress, which in turn resulted in a weakened immune system and a higher probability of successful pathogen transmission and zoonoses. In another scenario, a high burden of infectious diseases in the community compromised the immune system leading to increased physiological stress and metabolic disturbances. The cause-and-effect relationship is unclear and it is likely that multiple factors simultaneously contributed to the elevated stress in the Lauchheim community. Interestingly, Europe experienced a major climate decline between the fifth and seventh century CE, i.e., the Late Antique Little Ice Age (LALIA), which was likely an environmental driver of crop failure, famine, and disease [24]. Although the degree to which LALIA affected the Lauchheim community is unknown, it presents one possible explanation for the high disease burden. The far-reaching contacts and networks during that time, also documented for Lauchheim [25], might have contributed to the spread of infections.

It is notable that LALIA coincides with several changes in the pathogen landscape. A case in point is the Justinian plague (541-543 CE) that affected millions of people across Europe. Another example is that the dating of the G31 VARV genome together with VK388 and VK382 (Fig. 4A) falls within LALIA. Moreover, the early medieval *M. leprae* infections were the starting point of the leprosy epidemic during the eleventh–fourteenth centuries CE. This scenario is supported by the ancestral positions of the early Lauchheim (G83) and the British (GC96) genomes in clade 3 of the phylogeny (Fig. 5), which were to dominate the later epidemic (Additional file 1: Fig. S7) [23]. Taken together, we would like to hypothesize that LALIA may have created an ecological context in which persistent outbreaks set the stage for severe epidemics hundreds of years later. This speculation, however, needs corroboration.

The pathogens detected in Lauchheim are responsible for some of the most feared diseases of the last millennium and, with the exception of variola virus, still represent major health burdens today.

## Conclusions

Systematic pathogen screening of skeletal remains belonging to 70 individuals from the Lauchheim “Mittelhofen” provided molecular evidence for the high burden of infectious diseases in this early medieval community (> 30% of the individuals were positive for at least one pathogen at the time of death). Such a high frequency of infection together with physiological stress markers on the bones indicate an overall poor health status of the community that lived in a time period characterized by rapid cultural transition and major climate changes (e.g., LALIA). As seen today, climate change is an eminent driver of emergence, re-emergence and transmission of infectious diseases.

## Material

The material comprised bone and tooth samples of 70 individuals (Additional file 1: Tab. S1) excavated at the Lauchheim “Mittelhofen” settlement located in today’s Baden-Württemberg (southern Germany). The settlement was inhabited from the sixth to twelfth century CE (dating derived primarily from ceramic findings) [8, 26]. However, the settlement was used as a burial ground only during the late seventh and eighth century CE. Seventy-nine burials containing the remains of 77 individuals were found within the settlement borders [26].

## Methods

### Processing of samples

Processing of samples and DNA extraction were carried out in a dedicated ancient DNA facility at the University of Kiel. DNA extraction, library preparation, sequencing, and post-processing were performed according to a previously published protocol for non-UGD treated samples [27]. Subsequently, all generated metagenomic datasets were screened for the presence of pathogens with Megan Alignment Tool 0.3.0 (MALT) [28] (SemiGlobal alignment mode, identity threshold = 85%), using a custom database containing all bacterial and viral genomes from the NCBI website (download: 24.01.2019). Output alignments were inspected visually in MEGAN 6 [29]. Samples showing presence of pathogen DNA were further sequenced to obtain more data (Additional file 1: Tab. S3). Additionally, virus (111 human pathogens available on the NCBI website in 2018) targeted capture was performed on the VARV-positive sample (G31). Enriched data were then mapped against reference genomes of the respective pathogens to confirm the findings.

### *M. leprae* phylogeny

The pre-processed reads were mapped against the *M. leprae* TN reference genome (NC_002677) with the Burrows-Wheeler Aligner (BWA) v0.7.12 [30]. Output SAM files were converted to BAM format and merged, sorted and indexed with SAMtools v1.3 with default parameters [31]. Duplicated reads were removed using DeDup v0.12.2 [32] and the BAM file was filtered for quality 30. To generate VCF files, UnifiedGenotyper module from the Genome Analysis Toolkit (GATK) v3.6 was used. All the reads in the input were assigned to a single new read group with the Picard AddOrReplaceReadGroups tool with default parameters [33].

Based on a previously published dataset [21], 3124 SNPs with a minimum coverage of 3 x, a quality score of 30 and a 90% majority call were extracted. The phylogenetic *M. leprae* tree was produced with RaxML [34] with a bootstrap of 500 replicates (GTR- GAMMA model). The tree consisted of 176 previously published *M. leprae* strains (37 ancient and 139 modern) and the G83 individual. *M. lepromatosis* was used as an outgroup (Additional file 1: Tab. S10).

### HBV analysis

Using BWA [30], the HBV positive samples were mapped against a reference dataset representing the modern and known ancient diversity of HBV (Additional file 1: Tab. S11) as previously described [11]. BAM files belonging to one individual were merged using SAMtools [31], and duplicates were removed using the software DeDup with the default options [32]. BamUtil was used to clip two nucleotides from the 5′ and 3′ end. After a second round of duplicate removal, FASTQ was extracted from the BAM files using SAMtools. Subsequently a de novo assembly for HBV-positive samples was performed and resulting contigs were mapped (using BWA) against the respective sequence dataset containing all the references (Additional file 1: Tab. S11). For genomic reconstruction, the results of the remapping were visually inspected and, if possible, the consensus sequence was directly exported. Subsequent curation of the genomes was done manually. Using the Needleman-Wunsch algorithm, the ancient HBV genomes from Lauchheim were aligned to strains listed in the Additional file 1: Table S12.

A dataset containing 109 representative modern HBV genomes, 17 ancient HBV genomes and the four genomes from Lauchheim was used for the generation of a phylogenetic tree. A multiple sequence alignment was made using MAFFT [35] and phylogenetic informative blocks were extracted using GBLOCKS with default parameters [36]. A maximum likelihood phylogeny was generated using PhyML with the GTR substitution model, estimated proportion of invariable sites, NNI tree topology search, and 500 bootstrap replicates [37].

### B19 analyses

Parvovirus B19-positive samples were aligned against a dataset representing the modern and known ancient diversity of B19 diversity (Additional file 1: Tab. S13). Using samtools idxstats, AJ717293 was identified as the best-fitting reference, and subsequent mapping against AJ717293 was performed (Additional file 1: Tab. S14). Further processing of BAM files was done as described in the HBV section. For genomic reconstruction, the results of the mapping against AJ717293 were visually inspected and the consensus sequence was directly exported. Using the Needleman-Wunsch algorithm, the ancient B19 genome from Lauchheim was aligned to strains in the Additional file 1: Tab. S15. A MAFFT alignment followed by GBLOCKS was performed. A maximum likelihood phylogeny was generated using PhyML with the TN93 substitution model, estimated proportion of invariable sites, NNI tree topology search and 500 bootstrap replicates.

### Generation of VARV consensus sequence

Reads from individual G31 were mapped against the sequence of VK382 (10.6084/m9.figshare.12185466.v1) in Geneious Prime (https://www.geneious.com). The alignment was manually checked for insertions and deletions. The consensus sequence was called at 1 x coverage, if identical to the reference. SNPs were called if present in at least two reads.

### VARV gene loss and phylogenetic analyses

Gene loss analysis was performed as described [3]. For phylogenetic analyses, a multiple sequence alignment was constructed using available modern VARV and previously published ancient sequences [3, 38]. Only the central region of the genome (VACV-Cop *F4L* to VACV-Cop *A24R*) was used. A maximum likelihood (ML) tree was constructed and a regression of sampling dates and root-to-tip distances (extracted from the ML tree) was performed. Dating analyses were conducted with the same alignment that was used for the ML tree. A TPM1 substitution model with unequal base frequencies and invariant sites was selected, and the clock rate was constrained using a uniform distribution with bounds (1 × 10^−9^–10x^−3^ substitutions/site/year). After path sampling, likelihood values between different combinations of priors and clock models were compared with a Bayes factor test. Median node ages and substitution rates were inferred from trees run for 10M (for strict clock models) or 100M (for log-normal relaxed clock models) generations.

### Genetic sex determination

The genetic sex of the analyzed individuals was determined based on the ratio of sequences aligning to the X and Y chromosomes compared to the autosomes [39].

## Supplementary Information

Additional file 1: Supplementary figures S1-S5, and supplementary tables S1-S15.

Additional file 2. Review history.

## Data Availability

Analyzed sequences are available through the European Nucleotide Archive under Accession Number PRJEB49149: http://www.ebi.ac.uk/ena/browser/view/<accession> [40].
