# Supplementary material for: Pathogen genomics study of an early medieval community in Germany reveals extensive co-infections
Source: Genome Biol. 2022 Dec 13;23:250. doi: 10.1186/s13059-022-02806-8 (PMC9746117; doi:10.1186/s13059-022-02806-8)
Supplement: Supplementary file 1 — Additional file 1: Supplementary figures S1-S5, and supplementary tables S1-S15. [file 13059_2022_2806_MOESM1_ESM.docx]

ADDITIONAL FILE 1 – SUPPLEMENTARY INFORMATION

**Figure S1.** A maximum-likelihood tree illustrating the phylogenetic position of the HBV strains from Lauchheim (red).

Other 17 ancient are shown in blue and 109 modern strains in black. Numbers at each node are bootstrap support over 500 replications. Country codes can be found in Table S6. Dates are provided for ancient strains.

**Figure S2.** A maximum-likelihood tree illustrating the phylogenetic position of the B19 strain from Lauchheim (red).

Other 10 ancient strains are shown in blue and 77 modern strains in black. Numbers at each node are bootstrap support over 500 replications. Country codes can be found in Table S6. Dates are provided for ancient strains.

**Figure S3.** Gene inactivation patterns across ancient and modern VARVS.

The Lauchheim strain G31 is compared to previously published ancient and modern VARVs as well as the phylogenetically nearest zoonotic poxviruses: camelpox (CMLV) and taterapox (TATV). Colored circles indicate inactivated genes listed at the bottom of the figure.


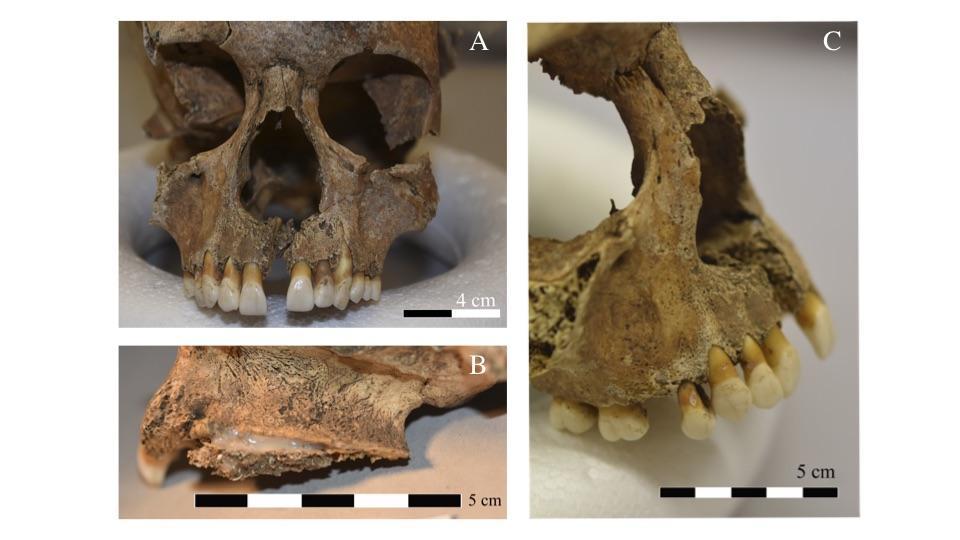


**Figure S4.** Individual G83 – likely leprosy-associated lesions of the cranium.

A - partial resorption of the anterior nasal spine accompanied by a widened nasal aperture with rounded margins; B - bilateral inflammation on the palatine process; C - microporosity (active inflammation process) on the anterior surface of the maxilla.

**Figure S5**. Individual G83 – lesions of the postcranial skeleton.

A - lytic lesions on the ventral surface of the manubrium; B - microporosity and haemorrhage on the 1^st^ left metatarsal; C - periostitis of the right distal tibia.

**Figure S6.** A maximum-likelihood tree illustrating the phylogenetic position of the *M. leprae* strain from Lauchheim (red). Other 37 ancient strains are shown in blue and 139 modern strains in black. Numbers at each node are bootstrap support over 500 replications. Country codes can be found in Table S6. Dates are provided for ancient strains.

**Figure S7.** Temporal and geographical distribution of medieval *M. leprae* strains.

**Table S1.** Sample characteristics^†^.

| **Grave No.** | **Sampled material** | **Age (years)** | **Sex** |
| --- | --- | --- | --- |
| 1 | tooth | 20-25 | male |
| 2 | petrous bone | 50-60 | male |
|  | tooth |  |  |
| 3 | tooth | 20-40 | female |
| 4 | tooth | 20-25 | male |
|  | petrous bone |  |  |
| 5 | tooth | 40-50 | male |
| 6 | petrous bone | 25-40 | female |
|  | tooth |  |  |
| 7 | tooth | approx. 25 | male |
| 8 | tooth | 50-60 | male |
| 9^‡^ | petrous bone | neonate | female |
| 10 | tooth | adult | male |
| 11 | tooth | adult | male |
| 13 | petrous bone | mature adult | female |
|  | tooth |  |  |
| 14 | petrous bone | 35-45 | female |
|  | tooth |  |  |
| 15 | petrous bone | 35-45 | male |
|  | tooth |  |  |
| 16^$^ | tooth | 20-25 | female |
|  | petrous bone |  |  |
| 21 | petrous bone | 19-21 | male |
|  | tooth |  |  |
| 22^‡^ | petrous bone | approx. 15 | male |
|  | tooth |  |  |
| 23 | tooth | 21-23 | male |
| 24 | petrous bone | senile | female |
|  | tooth |  |  |
| 25 | petrous bone | mature adult | male |
|  | tooth |  |  |
| 27^$^ | tooth | 30-35 | male |
| 28 | tooth | 30-40 | male |
|  | tooth |  |  |
| 29^$^ | tooth | 20-25 | female |
| 30 | tooth | mature/senile | male |
| 31^$^ | petrous bone | 25-30 | female |
|  | tooth |  |  |
| 33 | tooth | 40-50 | male |
| 35 | tooth | approx. 25 | female |
|  | petrous bone |  |  |
| 36^‡^ | petrous bone | 4 | male |
|  | tooth |  |  |
| 37 | petrous bone | approx. 25 | male |
|  | tooth |  |  |
| 38 | petrous bone | approx. 50 | female |
|  | tooth |  |  |
| 39 | tooth | 35-40 | male |
|  | petrous bone |  |  |
| 40^‡^ | tooth | 1.5-2 | indeterminate |
| 44 | tooth | 20-25 | female |
|  | petrous bone |  |  |
| 45 | petrous bone | adult | male |
|  | tooth |  |  |
| 46^‡,$^ | tooth | 12-14 | probable female^#^ |
|  | skull fragment |  |  |
| 47^‡^ | tooth | 15-18 | male |
| 48 | tooth | 20-25 | female |
|  | petrous bone |  |  |
| 49^‡^ | petrous bone | 2-3 | female |
| 50 | petrous bone | mature adult | female |
|  | tooth |  |  |
| 52^‡^ | petrous bone | 9-12 | female |
| 53 | tooth | 25-40 | male |
|  | petrous bone |  |  |
| 54 | petrous bone | mature/senile | male |
| 55 | petrous bone | 20-25 | male |
|  | tooth |  |  |
| 56 | petrous bone | mature adult | male |
|  | tooth |  |  |
| 57 | petrous bone | adult | indeterminate |
|  | tooth |  |  |
| 58 | petrous bone | adult | male |
|  | tooth |  |  |
| 59 | tooth | 30-40 | female |
|  | petrous bone |  |  |
| 60 | tooth | mature adult | female |
| 62 | petrous bone | 45-60 | male |
|  | tooth |  |  |
| 63 | tooth | 20-30 | male |
| 65 | petrous bone | approx. 25 | male |
| 66^‡,$^ | petrous bone | 6-8 | male |
|  | tooth |  |  |
| 67^‡^ | petrous bone | 16-20 | female |
|  | tooth |  |  |
| 68 | tooth | 30-40 | male |
|  | petrous bone |  |  |
| 69 | tooth | 40-50 | female |
| 70 | petrous bone | 30-40 | female |
|  | tooth |  |  |
| 71 | petrous bone | 45 < | female |
|  | tooth |  |  |
| 72^‡^ | petrous bone | 4-5 | male |
| 73 | tooth | 60-70 | female |
|  | petrous bone |  |  |
| 74 | tooth | 40-50 | male |
| 75 | petrous bone | approx. 50 | female |
| 77^‡^ | petrous bone | 9-11 | female |
| 78 | tooth | 40-50 | male |
| 79^‡,&^ | tooth | 13-14 | male |
|  | sternum |  |  |
| 80^‡^ | petrous bone | 13-15 | male |
| 82 | tooth | mature/senile | male |
| 83^‡, $^ | tooth | 13-15 | male |
|  | petrous bone |  |  |
| 84 | tooth | 20-25 | male |
| 85 | tooth | 30-35 | female |
| 86 | tooth | approx. 60 | male |

^#^- based on osteological data; ^‡^ - subadult; ^&^ - double infection; ^$^ - triple infection;

^†^ Seventy-nine burials with 77 individuals were found within the settlement borders. Due to poor preservation, no samples could be collected from 6 burials. In total, 71 individuals were sampled and aDNA was successfully retrieved for 70 individuals.

**Table S2.** Number of detected HBV and B19 reads in each Lauchheim sample.

Screening indicates the number of detected reads using MALT and mapping indicates the number of reads detected mapping to a specific reference (NC_003977.2 for HBV and NC_000883.2 for B19, number in brackets depicts the number of reads before min MQ=30 filtering). Screening and mapping were performed as described in the methods section. T = tooth, PB = petrous bone

| **Grave No.** | **Sampled**  **material** | **Number of**  **generated reads** | **HBV reads**  **screening** | **HBV reads**  **mapping** | **HBV reads**  **mapping2**^#^ | **B19 reads**  **screening** | **B19**  **mapping** | **B19**  **mapping2**^#^ |
| --- | --- | --- | --- | --- | --- | --- | --- | --- |
| **1** | T | 8350040 | - | - | - | 1 | 0(4) | 0(2) |
| **2** | PB | 7421840 | - | - | - | - | - | - |
|  | T | 8545724 | - | - | - | - | - | - |
| **3** | T | 9652938 | - | - | - | 7 | 0(0) | 0(6) |
| **4** | T | 11882300 | - | - | - | - | - | - |
|  | PB | 7585447 | - | - | - | - | - | - |
| **5** | T | 12504543 | - | - | - | 52 | 0(4) | 1(64) |
| **6** | PB | 5958210 | - | - | - | - | - | - |
|  | T | 1831669 | - | - | - | 1 | 0(0) | 0(1) |
| **7** | T | 11157115 | - | - | - | 124 | 0(1) | 5(136) |
| **8** | T | 17493206 | - | - | - | 25 | 1(3) | 1(30) |
| **9** | PB | 10723487 | - | - | - | - | - | - |
| **10** | T | 5120931 | - | - | - | - | - | - |
| **11** | T | 14269065 | - | - | - | 2 | 0(0) | 0(4) |
| **13** | PB | 5377322 | - | - | - | - | - | - |
|  | T | 8761379 | - | - | - | 3 | 0(0) | 0(3) |
| **14** | PB | 35337 | - | - | - | - | - | - |
|  | T | 8686209 | - | - | - | - | - | - |
| **15** | PB | 7619958 | - | - | - | - | - | - |
|  | T | 5712222 | - | - | - | - | - | - |
| **16** | T | 13569908 | 1 | 0(0) | 1(1) | 21 | 0(1) | 2(25) |
|  | PB | 4920577 | - | - | - | - | - | - |
| **21** | PB | 12223209 | - | - | - | - | - | - |
|  | T | 9879562 | - | - | - | - | - | - |
| **22** | PB | 6768458 | - | - | - | - | - | - |
|  | T | 14590353 | - | - | - | 280 | 1(15) | 9(294) |
| **23** | T | 10947354 | 2 | 0(0) | 0(0) | 212 | 1(8) | 6(243) |
| **24** | PB | 4265421 | - | - | - | - | - | - |
|  | T | 11383061 | - | - | - | 76 | 1(2) | 3(82) |
| **25** | T | 7662399 | - | - | - | 4 | 0(0) | 0(1) |
|  | PB | 10081594 | - | - | - | - | - | - |
| **27** | T | 12733614 | 13008 | 0(0) | 4215(4804) | 4483 | 0(0) | 181(3331) |
| **28** | T | 6771772 | - | - | - | 18 | 0(0) | 0(15) |
|  | T | 5357809 | - | - | - | - | - | - |
| **29** | T | 14938411 | 24 | 0(0) | 13(15) | 637 | 1(10) | 17(610) |
| **30** | T | 10467504 | - | - | - | 3 | 0(0) | 0(8) |
| **31** | T | 17356000 | - | - | - | 25 | 0(1) | 5(255) |
|  | PB | 5559428 | - | - | - | - | - | - |
| **33** | T | 6721479 | - | - | - | - | - | - |
| **35** | PB | 5630847 | - | - | - | - | - | - |
|  | T | 13770631 | - | - | - | - | - | - |
| **36** | PB | 9339543 | - | - | - | - | - | - |
|  | T | 10517234 | - | - | - | - | - | - |
| **37** | T | 8412554 | - | - | - | 23 | 1(2) | 2(29) |
|  | PB | 4529122 | - | - | - | - | - | - |
| **38** | PB | 8333303 | - | - | - | - | - | - |
|  | T | 4273011 | - | - | - | - | - | - |
| **39** | T | 7157365 | - | - | - | - | - | - |
|  | PB | 6431833 | - | - | - | - | - | - |
| **40** | T | 4627821 | - | - | - | - | - | - |
| **44** | T | 15657879 | - | - | - | - | - | - |
|  | PB | 13012275 | - | - | - | - | - | - |
| **45** | T | 7107892 | - | - | - | 2 | 0(0) | 0(3) |
|  | PB | 4498721 | - | - | - | - | - | - |
| **46** | T | 8122017 | 30 | 0(0) | 19(20) | 9 | 1(4) | 2(16) |
|  | SK | 42101430 | - | - | - | - | - | - |
| **47** | T | 7633463 | - | - | - | 2 | 0(0) | 0(2) |
| **48** | T | 9095745 | - | - | - | - | - | - |
|  | PB | 2874 | - | - | - | - | - | - |
| **49** | PB | 15754785 | - | - | - | - | - | - |
| **50** | PB | 6338023 | - | - | - | - | - | - |
|  | T | 5071828 | - | - | - | 31 | 0(0) | 0(38) |
| **52** | PB | 5950912 | - | - | - | 1 | 0(0) | 0(1) |
| **53** | T | 17461304 | - | - | - | - | - |  |
|  | PB | 45855716 | - | - | - | - | - | - |
| **54** | T | 22861458 | - | - | - | 1 | 0(0) | 0(1) |
| **55** | PB | 44398110 | - | - | - | - | - | - |
|  | T | 5334320 | - | - | - | 14 | 0(2) | 0(0) |
| **56** | PB | 7599222 | - | - | - | - | - | - |
|  | T | 56095757 | - | - | - | 72 | 0(8) | 20(1231) |
| **57** | PB | 14704505 | - | - | - | - | - | - |
|  | T | 7634523 | - | - | - | 3 | 0(0) | 0(3) |
| **58** | PB | 5107155 | - | - | - | - | - | - |
|  | T | 10609841 | - | - | - | - | - | - |
| **59** | T | 11289918 | 3 | 0(1) | 3(3) | 18 | 0(0) | 0(16) |
|  | PB | 6117665 | - | - | - | - | - | - |
| **60** | T | 9588298 | - | - | - | 52 | 0(0) | 0(58) |
| **62** | PB | 4589777 | - | - | - | - | - | - |
|  | T | 8239654 | - | - | - | - | - | - |
| **63** | T | 32335369 | - | - | - | 135 | 0(5) | 19(1132) |
| **65** | PB | 13398964 | - | - | - | 10 | 0(9) | 0(9) |
| **66** | PB | 11839709 | - | - | - | - | - | - |
|  | T | 12184923 | 11 | 2(2) | 2(2) | 111 | 0(0) | 1(62) |
| **67** | PB | 9952661 | - | - | - | - | - | - |
|  | T | 7662399 | - | - | - | 232 | 0(5) | 4(150) |
| **68** | T | 10597952 | - | - | - | - | - | - |
|  | PB | 7449942 | - | - | - | - | - | - |
| **69** | T | 10129081 | - | - | - | 66 | 0(1) | 2(83) |
| **70** | T | 9870604 | - | - | - | 135 | 0(1) | 5(151) |
|  | PB | 3504321 | - | - | - | - | - | - |
| **71** | T | 8305769 | - | - | - | 8 | 0(0) | 0(8) |
|  | PB | 10897155 | - | - | - | - | - | - |
| **72** | PB | 17750880 | - | - | - | - | - | - |
| **73** | PB | 7746112 | - | - | - | - | - | - |
|  | T | 16200789 | - | - | - | 31 | 0(0) | 0(34) |
| **74** | T | 10155137 | - | - | - | 21 | 0(14) | 0(41) |
| **75** | PB | 16588090 | - | - | - | - | - | - |
| **77** | PB | 16195168 | - | - | - | - | - |  |
| **78** | T | 393868776 | 1613 | 22(22) | 1163(1297) | 44 | 0(5) | 7(78) |
| **79** | ST | 11940143 | - | - | - | - | - | - |
|  | T | 13535439 | 4669 | 0(0) | 1874(2215) | 61 | 0(0) | 1(63) |
| **80** | PB | 5012075 | - | - | - | - | - | - |
| **82** | T | 11211337 | - | - | - | 18 | 0(0) | 1(18) |
| **83** | PB | 10416185 | - | - | - | - | - | - |
|  | T | 956507093 | 9075 | 66(66) | 5736(7088) | 4164 | 2(53) | 305(5149) |
| **84** | T | 21792847 | - | - | - | 6 | 0(1) | 1(8) |
| **85** | T | 7567577 | - | - | - | - | - | - |
| **86** | T | 17467738 | - | - | - | 178 | 0(0) | 14(157) |

^#^ - after enrichment;

**Table S3.** List of samples in which pathogen DNA was detected during the screening process using MALT. After deeper sequencing and/or enrichment, 22 samples were confirmed as pathogen-positive and genome reconstruction was possible for seven strains from five samples. Screening indicates the pathogen detected with MALT. Number of reads aligned is shown in brackets. Number of mapped reads2 shows the number of reads after enrichment: before (in brackets) and after quality filtering.

| **Grave** | **N of reads** | **Screening** | **N of reads2#** | **Pathogen presence** | **N of mapped reads** | **Genome** | **Duplication rate [%]** |
| --- | --- | --- | --- | --- | --- | --- | --- |
| **5** | 9702913 | B19 (52) | 12504543 | C | 1(64) | - | - |
| **69** | 7454249 | B19 (66) | 10129081 | C | 2(83) | - | - |
| **60** | 6908766 | B19 (52) | 9588298 | NC | - | - | - |
| **82** | 7187928 | B19 (18) | 11211337 | C | 1(18) | - | - |
| **16** | 8639558 | B19 (21), HBV(1) | 13569908 | C | 2(25),  1(1) | - | - |
| **29** | 7054690 | B19 (637), HBV (24) | 14938411 | C | 1(10), 13(15) | - | - |
| **31** | 7718890 | B19 (25), VARV (81) | 17356000^$^ | C | 5(255),  213124^†^ | VARV | 97 |
| **63** | 8242404 | B19 (135) | 32335369 | C | 9(1132) | - | - |
| **56** | 10980520 | B19 (72) | 56095757 | C | 20(1231) | - | - |
| **8** | 6836797 | B19 (25) | 17493206 | NC | - | - | - |
| **37** | 6836797 | B19 (23) | 8412554 | C | 2(29) | - | - |
| **84** | 8672984 | B19 (6) | 21792847 | NC | - | - | - |
| **70** | 7827092 | B19 (135) | 9870604 | C | 5(151) | - | - |
| **74** | 7711019 | B19 (21) | 10155137 | NC | - | - | - |
| **78** | 6883762 | B19 (44), HBV (1613) | 393868776 | C (only HBV) | 7(78), 1163(1297) | HBV | 18 |
| **46** | 6698956 | B19 (9), HBV (30) | 8122017 | C | 2(16), 19(20) | - | - |
| **86** | 7005516 | B19 (178) | 17467738 | C | 14(157) | - | - |
| **73** | 6377186 | B19 (31) | 16200789 | NC | - | - | - |
| **7** | 7476508 | B19 (124) | 11157115 | C | 5(136) | - | - |
| **79** | 8805306 | B19 (61), HBV (4669) | 13535439 | C | 1(63), 1874(2215) | HBV | 46 |
| **27** | 9832313 | B19 (4483), HBV (13008) | 12733614 | C | 181(3331), 4215(4804) | HBV | 61 |
| **59** | 6429141 | B19 (18), HBV (3) | 11289918 | NC | - | - | - |
| **83** | 8465765 | B19 (4164), HBV (9075),  *M. leprae* (1342) | 956507093 | C | 5736(7088), 305(5149), 252512 (394492) | B19, HBV, *M. leprae* | 29, 20, 25 |
| **24** | 7140143 | B19 (76) | 11383061 | C | 3(82) | - | - |
| **22** | 9228908 | B19 (280) | 14590353 | C | 9(294) | - | - |
| **71** | 4966852 | B19 (8) | 8305769 | NC | - | - | - |
| **66** | 8236574 | B19 (111), HBV (11) | 12184923 | C | 1(62), 2(2) | - | - |
| **13** | 6144837 | B19 (3) | 8761379 | NC | - | - | - |
| **6** | 5950635 | B19 (1) | 1831669 | NC | - | - | - |
| **25** | 6436574 | B19 (4) | 7662399 | NC | - | - | - |
| **67** | 2800683 | B19 (232) | 7662399 | C | 4(150) | - | - |
| **1** | 6711633 | B19 (1) | 8350040 | NC | - | - | - |
| **3** | 8545936 | B19 (7) | 9652938 | NC | - | - | - |
| **23** | 7965171 | B19 (212), HBV (2) | 10947354 | C (only B19) | 6(243) | - | - |
| **30** | 6227553 | B19 (3) | 10467504 | NC | - | - | - |
| **57** | 6625977 | B19 (3) | 7634523 | NC | - | - | - |
| **50** | 3704534 | B19 (31) | 5071828 | NC | - | - | - |
| **52** | 5950912 | B19 (1) | 5950912 | NC | - | - | - |
| **54** | 22861458 | B19 (1) | 22861458 | NC | - | - | - |
| **45** | 6339744 | B19 (2) | 7107892 | NC | - | - | - |
| **65** | 13398964 | B19 (10) | 13398964 | NC | - | - | - |
| **47** | 3628191 | B19 (2) | 7633463 | NC | - | - | - |
| **11** | 9213893 | B19 (2) | 14269065 | NC | - | - | - |
| **28** | 4662966 | B19 (18) | 6771772 | NC | - | - | - |

^#^ - after deeper shotgun sequencing; ^$^ - after virus capture; C – confirmed; NC – not confirmed; ^†^ - enrichment resulted in a 1200x increase of the number of VARV reads

**Table S4.** aDNA damage frequencies for the first two terminal bases of pathogen-positive samples that were used for further analysis.

| **Sample** | **Pathogen** | **3pGtoA pos1** | **3pGtoA pos2** | **5pCtoT pos1** | **5pCtoT pos2** |
| --- | --- | --- | --- | --- | --- |
| **G78** | HBV | 0.034 | 0.016 | 0.036 | 0.012 |
| **G79** | HBV | 0.056 | 0.023 | 0.031 | 0.013 |
| **G27** | HBV | 0.054 | 0.014 | 0.048 | 0.020 |
| **G83** | HBV | 0.048 | 0.011 | 0.055 | 0.013 |
| **G83** | B19 | 0.094 | 0.008 | 0.084 | 0.024 |
| **G83** | *M. leprae* | 0.028 | 0.012 | 0.03 | 0.014 |

**Table S5.** Characteristics of detected HBV and B19 genomes.

| **HBV** | | | | | | | | | | |
| --- | --- | --- | --- | --- | --- | --- | --- | --- | --- | --- |
| **Sample ID** | **GT** | **Seq. identity** | **No. of Ns** | | **GL** | **SER** | | **Insertions/ deletions** | **HBeAG** |  |
| G78 | D4/D4 | 97.1% | 10 | | 3182 | *ayw2* | | -/33-nt in *preS1* | positive |  |
| G79^‡^ | D4/D4 | 97.3% | 125 | | 3182 | *ayw2* | | -/33-nt in *preS1* | positive |  |
| G27 | D4/D4 | 97.6% | 11 | | 3182 | *ayw2* | | -/33-nt in *preS1* | positive |  |
| G83^‡^ | D4/D4 | 97.8% | 2 | | 3182 | *ayw2* | | -/33-nt in *preS1* | positive |  |
| **B19** | | | | | | | **NS1 and VP1/2 ORF completeness** | | | |
| G27 | - | - | 1520 | 4922 | | - | | | |  |
| G83^‡^ | 2/- | 96% | 132 | 4922 | | complete | | | |  |

^#^-predicted status; GT – genotype called by the two different approaches; Seq. identity – sequence identity to the closest modern reference EMBOSS; GL – genome length; SER – serotype; ^‡^- subadult

**Table S6.** Country codes.

| **Code** | **Country** | **Code** | **Country** | **Code** | **Country** |
| --- | --- | --- | --- | --- | --- |
| AF | Afghanistan | HK | Hong Kong | NI | Nicaragua |
| AR | Argentina | HT | Haiti | NO | Norway |
| AU | Australia | HU | Hungary | NP | Nepal |
| BD | Bangladesh | ID | Indonesia | PA | Panama |
| BY | Belarus | IN | India | PG | Papua New Guinea |
| BE | Belgium | IR | Iran | PH | Philippines |
| BJ | Benin | IQ | Iraq | PK | Pakistan |
| BO | Bolivia | IT | Italy | PL | Poland |
| BR | Brazil | JP | Japan | PT | Portugal |
| BW | Botswana | KG | Kyrgyzstan | RS | Serbia |
| CA | Canada | KH | Cambodia | RU | Russia |
| CG | Congo | KR | Korea | SE | Sweden |
| CH | Switzerland | KW | Kuwait | SK | Slovakia |
| CM | Cameroon | KZ | Kazakhstan | SL | Sierra Leone |
| CN | China | LB | Lebanon | SO | Somalia |
| CR | Costa Rica | LR | Liberia | SS | South Sudan |
| CZ | Czech Republic | LT | Lithuania | SY | Syria |
| DE | Germany | ML | Mali | TH | Thailand |
| DK | Denmark | MM | Myanmar | TR | Turkey |
| ET | Ethiopia | MN | Mongolia | TW | Taiwan |
| FI | Finland | MQ | Martinique | TZ |  |
| FR | France | MW | Malawi | US | United States of America |
| GB | Great Britain | MX | Mexico | VE | Venezuela |
| GL | Greenland | NC | New Caledonia | VN | Vietnam |
| GM | Gambia | NE | Netherlands | YE | Yemen |
| GN | Guinea | NG | Nigeria | ZA | South Africa |
| GR | Greece |  |  |  |  |

**Table S7.** Basic mapping statistics against the VK382 reference for the examined tooth sample G31. Screening indicates the number of detected reads using MALT.

| **N of reads**^#^ | **Screening**^#^ | **N of reads2**^‡^ | **No. mapped unique reads**^‡^ | **Mean coverage [%]** | **Depth** |
| --- | --- | --- | --- | --- | --- |
| 771889 | 81 | 17356000 | 213124 | 98 | 60.8 |

^#^  - before enrichment; ^‡^ - after enrichment

**Table S8.** Basic mapping statistics against the *M. leprae* TN reference for the examined tooth sample G83. Screening indicates the number of detected reads using MALT.

| **N of reads** | **Screening**^#^ | **N of mapped reads**^#^ | **N of mapped reads2**^‡^ | **Endogenous DNA [%]** | **Mean coverage** | **Coverage  1x [%]** | **Coverage  2x [%]** | **Coverage  3x [%]** |
| --- | --- | --- | --- | --- | --- | --- | --- | --- |
| 8465765^#^ | 1342 | 3813 | 394492 | 4.12 | 5.56 | 93.52 | 77.32 | 56.26 |
| 956507093^‡^ |  |  | 252512^§^ | 2.64 | 4.00 | 90.05 | 77.35 | 49.96 |

^#^  - before enrichment; ^‡^ - after enrichment; ^§^- filtered for quality of at least 30

**Table S9.** Skeletal lesions of individual G83.

| **CRANIAL SKELETON** | |
| --- | --- |
| **Lesion** | **Location** |
| Fibrous bone deposits  (*serpens endocrania symetrica*, SES) | Endocranial surface of the parietal bones and along the sagittal suture of the frontal bone |
| Partial bone resorption | Frontal nasal spine |
| Widening of the *apertura piriformis* accompanied by rounded margins | Nasal aperture |
| New bone formation | Palatine process and nasal facies of the maxilla |
| Active porotic lesions (*cribra orbitalia* and *cribra* *cranii*) | Orbital roof of the frontal bone and exocranial surface of the parietal bones |
| **POSTCRANIAL SKELETON** | |
| **Lesion** | **Location** |
| Active osteolytic lesions | Ventral surface of the manubrium |
| Evidence of haemorrhage | Shaft of the first and second left metatarsals |
| Microporosity | Shaft of the first right and second left metatarsals |
| Active periostitis | Medial and lateral surfaces of the tibae (the distal third of the shaft) |

**Table S10.** *M. leprae* strains implemented in the phylogenetic analysis.

| **Strain ID** | **Accession No.** | **Origin** | **Strain ID** | **Accession No.** | **Origin** |
| --- | --- | --- | --- | --- | --- |
| 1262-16 | [SRR6241805](https://www.ebi.ac.uk/ena/data/view/SRR6241805) | Pakistan | Kanazawa | [SRR6241778](https://www.ebi.ac.uk/ena/data/view/SRR6241778) | Japan |
| 2936 | [SRR6241802](https://www.ebi.ac.uk/ena/data/view/SRR6241802) | Malawi | Kitasato | [SRR6241779](https://www.ebi.ac.uk/ena/data/view/SRR6241779) | Japan |
| 2DDS | [SRR6241798](https://www.ebi.ac.uk/ena/data/view/SRR6241798) | Brazil | Korea-3-2 | [SRR6241784](https://www.ebi.ac.uk/ena/data/view/SRR6241784) | Korea |
| 85054 | [SRR6241803](https://www.ebi.ac.uk/ena/data/view/SRR6241803) | Martinique | Kusatsu-6 | [SRR6241785](https://www.ebi.ac.uk/ena/data/view/SRR6241785) | Japan |
| 97016 | [SRR6241804](https://www.ebi.ac.uk/ena/data/view/SRR6241804) | West Indies | Kyoto-1 | [SRR6241782](https://www.ebi.ac.uk/ena/data/view/SRR6241782) | Japan |
| Airaku2 | [SRR6241799](https://www.ebi.ac.uk/ena/data/view/SRR6241799) | Japan | Ml10-91 | [SRR6241783](https://www.ebi.ac.uk/ena/data/view/SRR6241776) | Mali |
| Airaku3 | [SRR1132821](https://www.ebi.ac.uk/ena/data/view/SRR1132821) | Japan | Ml10-93 | [SRR6241776](https://www.ebi.ac.uk/ena/data/view/SRR6241776) | Mali |
| Amami | [SRR6241800](https://www.ebi.ac.uk/ena/data/view/SRR6241800) | Japan | Ml10-94 | [SRR6241777](https://www.ebi.ac.uk/ena/data/view/SRR6241777) | Mali |
| ARLP_07 | [SRR6241801](https://www.ebi.ac.uk/ena/data/view/SRR6241801) | Ethiopia | Ml10-95 | [SRR6241793](https://www.ebi.ac.uk/ena/data/view/SRR6241793) | Mali |
| ARLP_08 | [SRR6241796](https://www.ebi.ac.uk/ena/data/view/SRR6241796) | Ethiopia | Ml10-96 | [SRR6241792](https://www.ebi.ac.uk/ena/data/view/SRR6241792) | Mali |
| ARLP_10 | [SRR6241797](https://www.ebi.ac.uk/ena/data/view/SRR6241797) | Ethiopia | Ml10-97 | [SRR6241791](https://www.ebi.ac.uk/ena/data/view/SRR6241791) | Mali |
| ARLP_11 | [SRR6241818](https://www.ebi.ac.uk/ena/data/view/SRR6241818) | Ethiopia | Ml10-98 | [SRR6241790](https://www.ebi.ac.uk/ena/data/view/SRR6241790) | Mali |
| ARLP_12 | [SRR6241819](https://www.ebi.ac.uk/ena/data/view/SRR6241819) | Ethiopia | Ml10-99 | [SRR6241789](https://www.ebi.ac.uk/ena/data/view/SRR6241789) | Mali |
| ARLP_13 | [SRR6241820](https://www.ebi.ac.uk/ena/data/view/SRR6241820) | Ethiopia | Ml2-10 | [SRR3330052](https://www.ebi.ac.uk/ena/data/view/SRR3330052) | Guinea |
| ARLP_14 | [SRR6241821](https://www.ebi.ac.uk/ena/data/view/SRR6241821) | Ethiopia | Ml2-5 | [SRR6241788](https://www.ebi.ac.uk/ena/data/view/SRR6241788) | Mali |
| ARLP_20 | [SRR6241822](https://www.ebi.ac.uk/ena/data/view/SRR6241822) | Ethiopia | Ml6-55 | [SRR3330054](https://www.ebi.ac.uk/ena/data/view/SRR3330054) | Guinea |
| ARLP_23 | [SRR6241823](https://www.ebi.ac.uk/ena/data/view/SRR6241823) | Ethiopia | Ml9-79 | [SRR6241787](https://www.ebi.ac.uk/ena/data/view/SRR6241787) | Mali |
| ARLP_25 | [SRR6241824](https://www.ebi.ac.uk/ena/data/view/SRR6241824) | Ethiopia | Ml9-80 | [SRR6241786](https://www.ebi.ac.uk/ena/data/view/SRR6241786) | Mali |
| ARLP_27 | [SRR6241825](https://www.ebi.ac.uk/ena/data/view/SRR6241825) | Ethiopia | Ml9-81 | [SRR6241795](https://www.ebi.ac.uk/ena/data/view/SRR6241795) | Mali |
| ARLP_29 | [SRR6241816](https://www.ebi.ac.uk/ena/data/view/SRR6241816) | Ethiopia | Ml9-82 | [SRR6241794](https://www.ebi.ac.uk/ena/data/view/SRR6241794) | Mali |
| ARLP_30 | [SRR6241817](https://www.ebi.ac.uk/ena/data/view/SRR6241817) | Ethiopia | Ml9-83 | [SRR6241806](https://www.ebi.ac.uk/ena/data/view/SRR6241806) | Mali |
| ARLP_32 | [SRR6241712](https://www.ebi.ac.uk/ena/data/view/SRR6241712) | Ethiopia | Ml9-84 | [SRR6241807](https://www.ebi.ac.uk/ena/data/view/SRR6241807) | Mali |
| ARLP_37 | [SRR6241711](https://www.ebi.ac.uk/ena/data/view/SRR6241711) | Ethiopia | Ml9-86 | [SRR6241808](https://www.ebi.ac.uk/ena/data/view/SRR6241808) | Mali |
| ARLP_40 | [SRR6241714](https://www.ebi.ac.uk/ena/data/view/SRR6241714) | Ethiopia | Ml9-87 | [SRR6241809](https://www.ebi.ac.uk/ena/data/view/SRR6241809) | Mali |
| ARLP_46 | [SRR6241713](https://www.ebi.ac.uk/ena/data/view/SRR6241713) | Ethiopia | Ng12-33 | [SRR6241810](https://www.ebi.ac.uk/ena/data/view/SRR6241810) | Niger |
| ARLP_48 | [SRR6241708](https://www.ebi.ac.uk/ena/data/view/SRR6241708) | Ethiopia | Ng13-32 | [SRR6241811](https://www.ebi.ac.uk/ena/data/view/SRR6241811) | Niger |
| ARLP_49 | [SRR6241707](https://www.ebi.ac.uk/ena/data/view/SRR6241707) | Ethiopia | Ng13-33 | [SRR6241812](https://www.ebi.ac.uk/ena/data/view/SRR6241812) | Niger |
| ARLP_52 | [SRR6241710](https://www.ebi.ac.uk/ena/data/view/SRR6241710) | Ethiopia | Ng14-35 | [SRR6241813](https://www.ebi.ac.uk/ena/data/view/SRR6241813) | Niger |
| ARLP_57 | [SRR6241709](https://www.ebi.ac.uk/ena/data/view/SRR6241709) | Ethiopia | Ng15-36 | [SRR6241814](https://www.ebi.ac.uk/ena/data/view/SRR6241814) | Niger |
| ARLP_62 | [SRR6241716](https://www.ebi.ac.uk/ena/data/view/SRR6241716) | Ethiopia | Ng16-38 | [SRR6241775](https://www.ebi.ac.uk/ena/data/view/SRR6241775) | Niger |
| ARLP_63 | [SRR6241715](https://www.ebi.ac.uk/ena/data/view/SRR6241715) | Ethiopia | Ng17-39 | [SRR6241774](https://www.ebi.ac.uk/ena/data/view/SRR6241774) | Niger |
| ARLP_65 | [SRR6241723](https://www.ebi.ac.uk/ena/data/view/SRR6241723) | Ethiopia | NHDP-55 | [SRR062543](https://www.ebi.ac.uk/ena/data/view/SRR062543) | USA |
| ARLP_68 | [SRR6241724](https://www.ebi.ac.uk/ena/data/view/SRR6241724) | Ethiopia | NHDP-63 | [SRR020801](https://www.ebi.ac.uk/ena/data/view/SRR020801) | USA |
| ARLP_73 | [SRR6241721](https://www.ebi.ac.uk/ena/data/view/SRR6241721) | Ethiopia | NHDP-98 | [SRR062638](https://www.ebi.ac.uk/ena/data/view/SRR062638) | USA |
| ARLP_74 | [SRR6241722](https://www.ebi.ac.uk/ena/data/view/SRR6241722) | Ethiopia | Oku-4 | [SRR6241773](https://www.ebi.ac.uk/ena/data/view/SRR6241773) | Japan |
| Bn7-39 | [SRR6241719](https://www.ebi.ac.uk/ena/data/view/SRR6241719) | Benin | Pak_A1 | [SRR6241772](https://www.ebi.ac.uk/ena/data/view/SRR6241772) | Pakistan |
| Bn7-41 | [SRR6241720](https://www.ebi.ac.uk/ena/data/view/SRR6241720) | Benin | Ryukyu-2 | [SRR6241771](https://www.ebi.ac.uk/ena/data/view/SRR6241771) | Japan |
| Bn8-46 | [SRR6241717](https://www.ebi.ac.uk/ena/data/view/SRR6241717) | Benin | S10 | [SRR847083](https://www.ebi.ac.uk/ena/data/view/SRR847083) | China |
| Bn8-47 | [SRR6241718](https://www.ebi.ac.uk/ena/data/view/SRR6241718) | Benin | S11 | [SRR847084](https://www.ebi.ac.uk/ena/data/view/SRR847084) | India |
| Bn8-51 | [SRR6241725](https://www.ebi.ac.uk/ena/data/view/SRR6241725) | Benin | S13 | [SRR847086](https://www.ebi.ac.uk/ena/data/view/SRR847086) | Mali |
| Bn8-52 | [SRR6241726](https://www.ebi.ac.uk/ena/data/view/SRR6241726) | Benin | S14 | [SRR847087](https://www.ebi.ac.uk/ena/data/view/SRR847087) | Mali |
| BP | [SRR6241730](https://www.ebi.ac.uk/ena/data/view/SRR6241730) | Brazil | S15 | [SRR847060](https://www.ebi.ac.uk/ena/data/view/SRR847060) | New Caledonia |
| BPA1 | [SRR6241730](https://www.ebi.ac.uk/ena/data/view/SRR6241730) | Brazil | S2 | [SRR847077](https://www.ebi.ac.uk/ena/data/view/SRR847077) | West Indies |
| Br1 | [SRR6241732](https://www.ebi.ac.uk/ena/data/view/SRR6241732) | Brazil | Thai-237 | [SRR6241770](https://www.ebi.ac.uk/ena/data/view/SRR6241770) | Thailand |
| Br14-1 | [SRR6241732](https://www.ebi.ac.uk/ena/data/view/SRR6241732) | Brazil | Thai-311 | [SRR6241769](https://www.ebi.ac.uk/ena/data/view/SRR6241769) | Thailand |
| Br14-2 | [SRR6241728](https://www.ebi.ac.uk/ena/data/view/SRR6241728) | Brazil | Thai53 | [SRR020800](https://www.ebi.ac.uk/ena/data/view/SRR020800) | Thailand |
| Br14-3 | [SRR6241727](https://www.ebi.ac.uk/ena/data/view/SRR6241727) | Brazil | TN | [AL450380.1](https://www.ncbi.nlm.nih.gov/nuccore/AL450380.1) | India |
| Br14-4 | [SRR6241734](https://www.ebi.ac.uk/ena/data/view/SRR6241734) | Brazil | Tsukuba-1 | [SRR6241768](https://www.ebi.ac.uk/ena/data/view/SRR6241768) | Japan |
| Br14-5 | [SRR6241733](https://www.ebi.ac.uk/ena/data/view/SRR6241733) | Brazil | US57 | [SRR6241767](https://www.ebi.ac.uk/ena/data/view/SRR6241767) | Marshall Islands |
| Br1A1 | [SRR6241732](https://www.ebi.ac.uk/ena/data/view/SRR6241732) | Brazil | W09 | [SRR062544](https://www.ebi.ac.uk/ena/data/view/SRR062544) | USA |
| Br2016-14 | [SRR6241731](https://www.ebi.ac.uk/ena/data/view/SRR6241731) | Brazil | Ye2-3 | [SRR6241766](https://www.ebi.ac.uk/ena/data/view/SRR6241766) | Yemen |
| Br2016-15 | [SRR6241736](https://www.ebi.ac.uk/ena/data/view/SRR6241736) | Brazil | Ye3s2 | [SRR6241737](https://www.ebi.ac.uk/ena/data/view/SRR6241737) | Yemen |
| Br2016-16 | [SRR6241735](https://www.ebi.ac.uk/ena/data/view/SRR6241735) | Brazil | Ye4-10 | [SRR6241751](https://www.ebi.ac.uk/ena/data/view/SRR6241751) | Yemen |
| Br2016-17 | [SRR6241745](https://www.ebi.ac.uk/ena/data/view/SRR6241745) | Brazil | Ye4-11 | [SRR6241752](https://www.ebi.ac.uk/ena/data/view/SRR6241752) | Yemen |
| Br2016-18 | [SRR6241746](https://www.ebi.ac.uk/ena/data/view/SRR6241746) | Brazil | Ye4-12 | [SRR6241753](https://www.ebi.ac.uk/ena/data/view/SRR6241753) | Yemen |
| Br2016-19 | [SRR6241747](https://www.ebi.ac.uk/ena/data/view/SRR6241747) | Brazil | Ye4-8 | [SRR6241754](https://www.ebi.ac.uk/ena/data/view/SRR6241754) | Yemen |
| Br2016-20 | [SRR6241748](https://www.ebi.ac.uk/ena/data/view/SRR6241748) | Brazil | Zensho-2 | [SRR6241755](https://www.ebi.ac.uk/ena/data/view/SRR6241755) | Japan |
| Br2016-21 | [SRR6241741](https://www.ebi.ac.uk/ena/data/view/SRR6241741) | Brazil | Zensho-4 | [SRR6241756](https://www.ebi.ac.uk/ena/data/view/SRR6241756) | Japan |
| Br2016-24 | [SRR6241742](https://www.ebi.ac.uk/ena/data/view/SRR6241742) | Brazil | Zensho-5 | [SRR6241757](https://www.ebi.ac.uk/ena/data/view/SRR6241757) | Japan |
| Br2016-26 | [SRR6241743](https://www.ebi.ac.uk/ena/data/view/SRR6241743) | Brazil | Zensho-9 | [SRR6241758](https://www.ebi.ac.uk/ena/data/view/SRR6241758) | Japan |
| Br2016-27 | [SRR6241744](https://www.ebi.ac.uk/ena/data/view/SRR6241744) | Brazil | 3077 | [SRR847038; SRR847039](https://www.ebi.ac.uk/ena/data/view/SRR847038) | Sweden |
| Br2016-45 | [SRR6241739](https://www.ebi.ac.uk/ena/data/view/SRR6241739) | Brazil | Body188 | [SRR6263249; SRR6263255](https://www.ebi.ac.uk/ena/data/view/SRR6263249) | Czech Republic |
| Br2016-46 | [SRR6241740](https://www.ebi.ac.uk/ena/data/view/SRR6241740) | Brazil | GC96 | [SRR6263252; SRR6263253; SRR6263254](https://www.ebi.ac.uk/ena/data/view/SRR6263253) | UK |
| Br2016-47 | [SRR6241759](https://www.ebi.ac.uk/ena/data/view/SRR6241759) | Brazil | Jorgen_1083 |  | Denmark |
| Br4923 | [GCF_000026685.1](https://www.ncbi.nlm.nih.gov/assembly/GCF_000026685.1) | Brazil | Jorgen_1149 |  | Denmark |
| BrMM1 | [SRR6241749](https://www.ebi.ac.uk/ena/data/view/SRR6241749) | Brazil | Jorgen_154 |  | Denmark |
| BrMM2 | [SRR6241761](https://www.ebi.ac.uk/ena/data/view/SRR6241761) | Brazil | Jorgen_34 |  | Denmark |
| BrMM4 | [SRR6241760](https://www.ebi.ac.uk/ena/data/view/SRR6241760) | Brazil | Jorgen_404 |  | Denmark |
| BrMM5 | [SRR6241763](https://www.ebi.ac.uk/ena/data/view/SRR6241763) | Brazil | Jorgen_427 |  | Denmark |
| Brw15_10 | [SRR3672752](https://www.ebi.ac.uk/ena/data/view/SRR3672752) | UK | Jorgen_507 |  | Denmark |
| Brw15-1E | [SRR3672737](https://www.ebi.ac.uk/ena/data/view/SRR3672737) | UK | Jorgen_533 |  | Denmark |
| Brw15-12M | [SRR3672753](https://www.ebi.ac.uk/ena/data/view/SRR3672753) | UK | Jorgen_625 |  | Denmark |
| Brw15-20M | [SRR3672757](https://www.ebi.ac.uk/ena/data/view/SRR3672757) | UK | Jorgen_722 |  | Denmark |
| Brw15-25E | [SRR3672758](https://www.ebi.ac.uk/ena/data/view/SRR3672758) | UK | Jorgen_749 |  | Denmark |
| Brw15-5E | [SRR3672738](https://www.ebi.ac.uk/ena/data/view/SRR3672738) | UK | Refshale_16 | [SRR847056; SRR847057](https://www.ebi.ac.uk/ena/data/view/SRR847056) | Germany |
| EGG | [SRR6241762](https://www.ebi.ac.uk/ena/data/view/SRR6241762) | Mexico | SK11 | [SRR6263250; SRR6263256](https://www.ebi.ac.uk/ena/data/view/SRR6263250) | Denmark |
| Fio3 | [SRR6241765](https://www.ebi.ac.uk/ena/data/view/SRR6241765) | Brazil | SK2 | [SRR7608163](https://www.ebi.ac.uk/ena/data/view/SRR7608163) | Hungary |
| Gu4-17 | [SRR6241764](https://www.ebi.ac.uk/ena/data/view/SRR6241764) | Guinea | SK8 | [SRR847069; SRR847070](https://www.ebi.ac.uk/ena/data/view/SRR847069) | UK |
| Gu5-23 | [SRR6241750](https://www.ebi.ac.uk/ena/data/view/SRR6241750) | Guinea | SK14 | [SRR6233936; SRR847036; SRR847064](https://www.ebi.ac.uk/ena/data/view/SRR6233936) | UK |
| Indonesia-1 | [SRR6241780](https://www.ebi.ac.uk/ena/data/view/SRR6241780) | Indonesia | T18 | [SRR6263251; SRR6263257](https://www.ebi.ac.uk/ena/data/view/SRR6263251) | UK |
| R7546-671 | SRR15512562  SRR15512564  SRR15512682  SRR15512683 | Russia | UF703 | SRR15512702  SRR15512703  SRR15512704  SRR15512705 | Spain |
| UF11 | SRR15512654  SRR15512655  SRR15512657  SRR15512658 | Spain | KirkHill | SRR15512519  SRR15512520  SRR15512522  SRR15512692  SRR15512693  SRR15512694  SRR15512695  SRR15512696  SRR15512697 | UK |
| UF21 | SRR15512648  SRR15512649  SRR15512650  SRR15512651  SRR15512652 | Spain | UF803 | SRR15512609  SRR15512610  SRR15512611 | Spain |
| UF25 | SRR15512646  SRR15512647 | Spain | CHRY044 | SRR15512532  SRR15512723 | UK |
| JDS097 | SRR15512689 | UK | BEL024 | SRR15512724 | Belarus |
| PAVd’09_I.5 | SRR15512575  SRR15512576  SRR15512577  SRR15512578  SRR15512579  SRR15512580  SRR15512581  SRR15512583  SRR15512584  SRR15512585  SRR15512586  SRR15512587  SRR15512588  SRR15512589  SRR15512590  SRR15512591  SRR15512592 | Portugal | CHRY023 | SRR15512549 | UK |
| Bergen | SRR15512594  SRR15512595 | Norway | EDI006 | SRR15512522  SRR15512521 | UK |
| UF700 | SRR15512713  SRR15512714  SRR15512715  SRR15512716  SRR15512717 | Spain | UF101 | SRR15512636  SRR15512637  SRR15512639  SRR15512638  SRR15512640 | Spain |
| Izumi | [SRR6241781](https://www.ebi.ac.uk/ena/data/view/SRR6241781) | Japan | *M. lepromatosis* | [SRR1576832](https://www.ebi.ac.uk/ena/data/view/SRR1576832) | Mexic  o |

**Table S11.** List of references used in the in the multi fasta file for the HBV mapping.

| **Accession** | **Genotype** | **Host** |
| --- | --- | --- |
| X51970 | A | Human |
| AB073846.1 | B | Human |
| M12906 | C | Human |
| M32138 | D | Human |
| AB032431 | E | Human |
| AB036910 | F | Human |
| AB064310 | G | Human |
| AY090454 | H | Human |
| LT992440.1 | A | Ancient Human Strain |
| LT992441.1 | A | Ancient Human Strain |
| LT992442.1 | B | Ancient Human Strain |
| LT992447.1 | A | Ancient Human Strain |
| LT992448.1 | A | Ancient Human Strain |
| LT992438.1 | D | Ancient Human Strain |
| LT992439.1 | D | Ancient Human Strain |
| LT992443.1 |  | Ancient Monkey Strain |
| LT992444.1 | D | Ancient Human Strain |
| LT992454.1 | D | Ancient Human Strain |
| LT992459.1 |  | Ancient Monkey Strain |
| JN315779.1 | C | Ancient Human Strain |
| MG585269.1 | D | Ancient Human Strain |
| Karsdorf |  | Ancient Monkey Strain |
| Sorsum |  | Ancient Monkey Strain |
| Petersberg | D | Ancient Human Strain |
| AB032433 |  | Chimpanzee |
| AF222323 |  | Chimpanzee |
| AJ131567 |  | Gorilla |
| AY330911.1 |  | Chimpanzee |
| AJ131571.1 |  | Gibbon |
| U46935.1 |  | Gibbon |
| FM209516.1 |  | Gibbon |
| AF193863.1 |  | Orangutan |

**Table S12.** Accession numbers for 17 ancient and 109 modern strains used in the phylogenetic analysis of HBV.

| **Accession numbers** | | | | | | |
| --- | --- | --- | --- | --- | --- | --- |
| LT992440.1 | MG585269.1 | AB032433 | AY781180 | AF193863 | AY902768 | AY738142 |
| LT992441.1 | PRJEB24921 (Karsdorf, Petersberg, Sorsum) | AB033558 | EU155824 | DQ315779 | GQ477453 | GQ477499 |
| LT992442.1 | FN545831 | GQ205378 | HE974378 | AM494716 | X75657 | AP011108 |
| LT992447.1 | EU859952 | GQ205382 | AB048702 | FJ904436 | X75664 | X75656 |
| LT992448.1 | KP322602 | GQ205385 | AB033559 | FJ904430 | GQ331046 | X75665 |
| LT992438.1 | KP322603 | GQ205389 | AB048701 | AB188243 | AY934764 | AP011102 |
| LT992439.1 | KC875319 | AY090455 | GQ922005 | JN688710 | GQ161813 | AP011103 |
| LT992443.1 | KP341007 | GQ205377 | JN642160 | FJ899792 | FJ692611 | AP011106 |
| LT992444.1 | KP322600 | GQ205384 | JN642163 | JN642140 | FJ692598 | AB111946 |
| LT992454.1 | FM209516 | AY330911 | AB210818 | JN688711 | FJ692556 | DQ089767 |
| LT992459.1 | AF222323 | AJ131567 | GQ477455 | KJ470898 | AB076679 | AB112472 |
| LT992455.1 | GQ331047 | U46935 | X80925 | KJ470893 | AB116084 | AB112066 |
| JN315779.1 | KC774243 | AJ131571 | AY796031 | KJ470896 | AB453988 | AF241411 |
| AP011100 | AB033555 | AB073835 | AB287321 | X75663 | AB116654 | AB241117 |
| AB048705 | AP011089 | DQ463792 | HE981175 | AB116549 | FJ657525 | DQ993686 |
| AB048704 | AB219430 | DQ463789 | AB486012 | DQ899144 | AB059660 | AB287318 |
| AB073858 | AB219429 | AB287316 | AF223962 | DQ899146 | AB375163 | AB166850 |
| AY311369 | AY090458 | AY090457 | AY090454 |  |  |  |

**Table S13.** List of references used in the in the multi-fasta file for the B19 mapping.

| **Accession** | **Genotype** |
| --- | --- |
| FN669502.1 | 1 |
| DQ357065.1 | 1 |
| AF113323.1 | 1 |
| AJ717293.1 | 2 |
| DQ333427.1 | 2 |
| HQ340602.1 | 2 |
| AY083234.1 | 3 |
| AJ249437.1 | 3 |
| NC_001540.1 | Bovine parvovirus |
| DA251 | 1 |
| DA337 | 1 |
| DA336 | 1 |
| DA66 | 1 |
| NEO105 | 1 |
| VK477 | 2 |
| VK143 | 2 |
| RISE569 | 2 |
| VK6 | 2 |
| VK154 | 2 |

**Table S14.** Number of reads mapping against the B19 multi-fasta reference and against the single genome AJ717293.

| **Sample** | **No. reads mapping to multi-fasta reference** | **No. reads mapping to AJ717293** |
| --- | --- | --- |
| **G27** | 1633 | 1329 |
| **G83** | 3536 | 2473 |

**Table S15.** Accession numbers for complete parvovirus B19 genomes used for genotype assignment and phylogenetic analysis.

| **Accession Number** | | | | | | |
| --- | --- | --- | --- | --- | --- | --- |
| AJ781038 | KM393165 | KM393168 | KM393166 | AB126267 | AB126266 | KM065415 |
| KC013340 | KT310174 | DQ225150 | DQ225149 | KR005641 | KR005640 | AY386330 |
| KM393163 | M13178 | FN598218 | Z70560 | Z68146 | DQ408301 | AB030673 |
| KM393164 | KC013329 | KC013325 | KT268312 | KC013305 | AF113323 | Z70599 |
| AY504945 | AB126271 | AB126262 | AB126269 | AF162273 | FJ591158 | KC013343 |
| KC013324 | KC013308 | KM393169 | AB030694 | DQ293995 | KC013316 | DQ225151 |
| KC013321 | KC013312 | KC013333 | KC013346 | KC013313 | KC013327 | AB126265 |
| AB126270 | DQ357065 | DQ357064 | KF724387 | AY903437 | AY044266 | DQ333426 |
| AB550331 | EF216869 | AJ717293 | KF724386 | DQ333428 | AY064476 | AY064475 |
| AY647977 | AY083234 | AY582124 | DQ234779 | DQ234778 | DQ408305 | DQ408302 |
| DQ408304 | DQ408303 | FJ265736 | DQ234775 | DQ234771 | DQ234769 | AJ249437 |
| DA251 | DA336 | DA337 | RISE569 | NEO105 | VK6 | VK143 |
| VK154 | VK477 | DA66 | DQ225149 | KR005641 | KR005640 | DQ225149 |
| AJ781038 | KM393165 | KM393168 | KM393166 | AB126267 | AB126266 | KM065415 |
| KC013340 | KT310174 | DQ225150 | |  |  |  |
