# Supplementary material for: Pathogen genomics study of an early medieval community in Germany reveals extensive co-infections
Source: Genome Biol. 2022 Dec 13;23:250. doi: 10.1186/s13059-022-02806-8 (PMC9746117; doi:10.1186/s13059-022-02806-8)
Supplement: Supplementary file 2 — Additional file 2. Review history. [file 13059_2022_2806_MOESM2_ESM.docx]

**1^st^ round**

**Reviewer 1**

This work is of broad interest to others in the field of infectious disease and of biologists. It represents an advance in that this is a cemetery-wide screening approach for an early medieval site and a very interesting set of data. The methods are appropriate to the aims of the study and are well described. The conclusions are adequately supported by the data and the details allow for replication and comparison. It would be nice to see how the M. leprae strain compares the the Edix Hill strain (500-650 CE) included in Pfrengle et al. 2021, however, I wouldn't require the inclusion of that data as a requisite for publication.

**Reviewer 2**

This is an interesting paper that describes the pathogen landscape of a rural burial dating between the 5th and 8th century. Amazingly 30% of the samples showed the presence of the DNA of various infectious agents. While the paper is fascinating and the methods solid, I have several minor and a few major issues that require addressing before this manuscript would be acceptable for publication.

One of the overwhelming narratives of the paper is to place these pathogens into a context regarding the pathogen landscape of a particular place and time. While these results do this, they cannot be linked necessarily to overlapping distributions, given the fact that the cemetery itself is in use for nearly three centuries. This doesn't minimize what the authors have found, but they must be careful to not ascribe this to one moment in time, this is a 300-year window that they are looking at, within which landscapes can change dramatically.

In the SOM - there is no discussion of the MALT identification of VARV or M. leprae. Can these data be added to show how the authors discovered these reads?

There is no discussion on the enrichment success of failure. What fold enrichments were obtained. Please show the stats for the shotgun vs enrichment for each of the samples that were ultimately enriched.

The table in SOM doesn't show which samples were the double infected individuals? Were the authors able to enrich and sequence the genomes of all pathogens in the case of doubly infected people? If so, it was not made immediately clear.

While I applaud the authors for using CPP as a way to assess the pathogen burden - please see my point above about the timing of all individuals. If the authors which to persue this line of inquiry and thesis, they need to provide a much better analysis and figure of the dates of each sample which contains pathogen DNA. This would entail Archaeological dates for each burial as well as carbon 14 dates and in the cases where molecular clocks are robust (VARV, M.L), tip dates estimated from calibrated rates. Currently there is no evidence to believe the CPP rate was 31.4% at the same point in time.

Again, please provide all stats for the enrichments (reads, duplication rate, etc.) of each sample. Since the authors have the shotgun data they used for screening this should be too onerous.

The long -term persistence of HBV (as subtype D) is interesting, may be worth include https://doi.org/10.1371/journal.ppat.1006750 - both into the context and on the tree. It shows long term continuity of the same strains circulating in Western Europe.

Line 161-163. The authors assume a direct transmission of HBV between two individuals. However given that we have no idea what the circulating diversity of HBV was at that time this is intriguing but cannot be certain. I'm not convinced that kin-analysis would necessarily clarify this point.

The VARV data is intriguing. The authors should be careful to distinguish these early circulating strains that lack the full complement of inactivated genes that define -from what we call Smallpox today. Despite that caveat it's interesting to see the diversity of gene inactivation when compared to the other 'viking' strains previously described. I think the authors could take a bit more time to unpack these data. They are correct to point out that if these strains were as infective as smallpox we identify today and they don't find anyone else harboouring the infection, doesn't that say something about it's potential transmissibility back then? This requires knowing again certain details about other burials that are co-occuring events. It may be that the authors don't have enough data to say much about the specifics of these findings.

While I like the archaeological work that was done to assess the skeletal remains for lesions - something that should be much more front and centre in this manuscript, the authors are again slightly stretching their interpretations by suggesting that evidence of stress lead to compromised immune systems and that in turn led to the higher prevelance of infectious disease at this site. Large scale sweeping statements about climate change - in this case the LALIA - are interesting and catchy but they are hard to normalize into an actual effect. They are difficult to test since we don't have specific dates here for individuals and we don't really know to what degree this particular population may have been affected by these major events. While I do like the narrative overall, I think the authors should tread lightly on making this a make or break case of the impact of the LALIA. The linking of measles emergence to this event is also over drawn. The authors here are simply linking the tMRCA of modern measles with rinderpest. But we have no idea when along that branch measles actually emerged (line 241).

The authors also point out the fact that the cementery abuts the Justinian plague, so it was surprising that no of the individuals had plague in them, is that true?

The authors cite a reference (12) for all their methods, but they need to include those details or some of them here.

Finally the authors mention that there was no need for ethics approval for their destructive analyses, can they describe why that's the case in this situation?

With these issues resolved the manuscript will make a worthy contribution to the field of ancient pathogenomics.

**Authors’ response**

Reviewer 1:

1)It would be nice to see how the M. leprae strain compares the the Edix Hill strain (500-650 CE) included in Pfrengle et al. 2021, however, I wouldn't require the inclusion of that data as a requisite for publication.

Response: The Edix Hill strain, together with 15 later-dating M. leprae genomes published by Pfrengle et al., 2021, was included in the phylogenetic analysis (Fig. 5) and in the supplementary map (Fig. S7). The difference between the Lauchheim and Edix Hill strains was mentioned in the discussion.

Lines 271-274 on page 13 in the manuscript:

Two British strains dating to a similar time period were included in the phylogenetic analysis (GC96, 5-6th century and EDI006, 6-7th century AD). The Lauchheim strain groups in branch 3 together with both strains, suggesting low diversity of M. leprae in the early medieval period [23].

Reviewer 2:

1)One of the overwhelming narratives of the paper is to place these pathogens into a context regarding the pathogen landscape of a particular place and time. While these results do this, they cannot be linked necessarily to overlapping distributions, given the fact that the cemetery itself is in use for nearly three centuries. This doesn't minimize what the authors have found, but they must be careful to not ascribe this to one moment in time, this is a 300-year window that they are looking at, within which landscapes can change dramatically.

Response: We would like to point out that the Merovingian period lasted for approx. 300 years. However, the period of the Lauchheim settlement burials was estimated with C14 dates of 7 infection-positive individuals, dendrochronology (Tab. 1) and archaeological artefacts to be around one century (mid 7th-mid 8th cent. AD). Following the reviewer’s advice, we clarified in the result section that the CPP was calculated for a period of approx. three to four generations and was not ascribed to one moment in time.

Lines 104-106 on page 5 in the manuscript:

CPP is defined as the percentage of individuals in a population (%) that exhibit a molecular sign of infection over the period of time in which the settlement was used as a burial ground (approx. a century). Thus, the CPP indicates the prevalence of infections over a period of about three to four generations, during which the pathogen landscape can change.

2)In the SOM - there is no discussion of the MALT identification of VARV or M. leprae. Can these data be added to show how the authors discovered these reads?

Response: Information on the number of VARV and M. leprae reads from mapping and/or pathogen screening before and after enrichment is now included in the SOM (Tables S6 and S7, respectively). For HBV and B19, this information can be found in Table S2). In the expanded methods section, a description of the pathogen identification process was added.

Lines 360-368 on pages 17-18 in the manuscript:

Subsequently, all generated metagenomic datasets were screened for the presence of pathogens with Megan Alignment Tool 0.3.0 (MALT) [28] (SemiGlobal alignment mode, identity threshold = 85%), using a custom database containing all bacterial and viral genomes from the NCBI website (download: 24.01.2019). Output alignments were inspected visually in MEGAN 6 [29]. Samples showing presence of pathogen DNA were further sequenced to obtain more data (Additional file 1: Tab. S3). Additionally, virus (111 human pathogens available on the NCBI website in 2018) targeted capture was performed on the VARV-positive sample (G31). Enriched data was then mapped against reference genomes of the respective pathogens to confirm the findings.

3)There is no discussion on the enrichment success of failure. What fold enrichments were obtained. Please show the stats for the shotgun vs enrichment for each of the samples that were ultimately enriched.

Response: This information is now included in the SOM (new Table S3). Number of reads in the initial shotgun sequenced samples and the enriched sample G31 are provided, together with duplication rates for the samples in which pathogen genomes were successfully reconstructed. Mapping statistics for detected pathogens are also provided in tables S7 (VARV), S8 (M. leprae) and S2 (HBV and B19). The enrichment factor for the VARV-positive sample was calculated (Tab. S3: “† - enrichment resulted in a 1200x increase of the number of VARV reads”). No enrichment was performed for B19, HBV and M. leprae-positive samples.

4)The table in SOM doesn't show which samples were the double infected individuals? Were the authors able to enrich and sequence the genomes of all pathogens in the case of doubly infected people? If so, it was not made immediately clear.

Response: Double and triple infected individuals are now marked in Table S1 in the SOM ( & - double infection; $ - triple infection) Enrichment of the samples from individuals infected by multiple pathogens did not result in successful genome reconstruction for all pathogens detected in these samples. It is now clarified in the results section and can also be seen in Table 1.

Lines 99-101 on page 5 in the manuscript:

Data enrichment allowed to reconstruct and analyse four HBV (G78, G79, G27, and G83) and one B19 (G83) genomes as well as the VARV (G31) and M. leprae genomes (G83) (Tab. 1)

5)While I applaud the authors for using CPP as a way to assess the pathogen burden - please see my point above about the timing of all individuals. If the authors which to persue this line of inquiry and thesis, they need to provide a much better analysis and figure of the dates of each sample which contains pathogen DNA. This would entail Archaeological dates for each burial as well as carbon 14 dates and in the cases where molecular clocks are robust (VARV, M.L), tip dates estimated from calibrated rates. Currently there is no evidence to believe the CPP rate was 31.4% at the same point in time.

Response: this issue was addressed for point 1), please see the answer above.

6)Again, please provide all stats for the enrichments (reads, duplication rate, etc.) of each sample. Since the authors have the shotgun data they used for screening this should be too onerous.

Response: this issue was addressed for point 3), please see the answer above.

7)The long -term persistence of HBV (as subtype D) is interesting, may be worth include https://doi.org/10.1371/journal.ppat.1006750 - both into the context and on the tree. It shows long term continuity of the same strains circulating in Western Europe.

Response: The 16th century HBV strain is now included in the tree (Fig. 2) and also mentioned in the Discussion section. Patterson Ross et al., 2018 was added to the reference list.

Lines 216-221 on page 11 in the manuscript:

All HBV genomes were of sub-genotype D4 – as previously found in 11th-12th century Petersberg (Germany) [11]. Today, sub-genotype D4 is mainly found in Australasia and Northern America. Thus, the available data point to a temporal change in the geographical distribution of HBV genotype D sub-genotypes in Europe – with early medieval D4 subtypes being replaced with those that are prevalent today, such as D1, D2, and D3 [12]. Interestingly, in 16th century Italy, an HBV strain of genotype D3 was already present [13] (Fig. 2).

8)Line 161-163. The authors assume a direct transmission of HBV between two individuals. However given that we have no idea what the circulating diversity of HBV was at that time this is intriguing but cannot be certain. I'm not convinced that kin-analysis would necessarily clarify this point.

Response: We agree with the reviewer’s observation and this section of the manuscript was modified:

Lines 223-227 on pages 11-12 in the manuscript:

Although the diversity of HBV at the time is unknown, this finding could possibly suggest a direct transmission between these individuals or a common source of infection.

9)The VARV data is intriguing. The authors should be careful to distinguish these early circulating strains that lack the full complement of inactivated genes that define -from what we call Smallpox today. Despite that caveat it's interesting to see the diversity of gene inactivation when compared to the other 'viking' strains previously described. I think the authors could take a bit more time to unpack these data. They are correct to point out that if these strains were as infective as smallpox we identify today and they don't find anyone else harboouring the infection, doesn't that say something about it's potential transmissibility back then? This requires knowing again certain details about other burials that are co-occuring events. It may be that the authors don't have enough data to say much about the specifics of these findings.

Response: Thank you for your suggestions. We have changed the wording in the discussion to emphasize the difference between the ancient and modern VARVs.

Lines 243-246 on page 12 in the manuscript:

In the phylogenetic analysis, the G31 strain clustered within the known diversity of early medieval VARVs from northern and eastern Europe [3]. These VARVs are distinguished from modern VARVs by a larger number of intact genes.

We have also added an additional sentence at the end of the results paragraph on VARV to highlight commonalities of gene loss patterns in G31 with the VK382-VK388 and VK281-VK470 branches.

Lines 160-164 on pages 8-9 in the manuscript:

Concerning the genes showing differences in activation patterns across the two branches, G31 shares gene-inactivating mutations with the VK382-VK388 branch in three genes (A39R, C2L, F3L) and with the VK281-VK470 branch in four genes (22682/CPXV020, K1L, 168145/CPXV181, A57R).

We agree with the reviewer that it would be good to say something about transmissibility, but as the reviewer says, further information would be required for this to be reliable, such as knowledge about the likelihood for the preservation of viral DNA in victims of the ancient poxvirus. Unfortunately, we do not have additional information that would allow us to make inferences about transmissibility, in fact even today it is difficult to do solid work on SARS-CoV-2 transmissibility despite the availability of vastly more data. The issues of prevalence and transmissibility are further clouded by the fact that (modern, at least) VARV is not viraemic throughout the infection, so additional undetectable cases may also have been present in the graveyard. Therefore, the absence of additional findings of ancient VARV in Lauchheim cannot form the basis of reliable conclusions regarding the transmissibility of the virus.

10)While I like the archaeological work that was done to assess the skeletal remains for lesions - something that should be much more front and centre in this manuscript, the authors are again slightly stretching their interpretations by suggesting that evidence of stress lead to compromised immune systems and that in turn led to the higher prevelance of infectious disease at this site.

Response: The interpretation of the osteological findings and the link with infectious diseases was revised according to the reviewer’s comment.

Lines 296-312 on pages 14-15 in the manuscript:

Interestingly, 95.5% of pathogen-positive individuals (Tab. 1) and 84.4% of individuals with no molecular sign of infection exhibited skeletal lesions suggestive of physiological stress. This extremely high burden of lesions indicates poor health in Lauchheim. In one scenario, malnutrition could have increased the level of physiological stress, which in turn resulted in a weakened immune system and higher probability of successful pathogen transmission and zoonoses. In another scenario, high burden of infectious diseases in the community compromised the immune system leading to increased physiological stress and metabolic disturbances. The cause-and-effect relationship is unclear and it is likely that multiple factors simultaneously contributed to the elevated stress in the Lauchheim community. Interestingly, Europe experienced a major climate decline between the 5th and 7th century AD, i.e., the Late Antique Little Ice Age (LALIA), which was likely an environmental driver of crop failure, famine, and disease [24]. Although the degree as to which LALIA affected the Lauchheim community is unknown, it presents one possible explanation for the high observed disease burden.

11) Large scale sweeping statements about climate change - in this case the LALIA - are interesting and catchy but they are hard to normalize into an actual effect. They are difficult to test since we don't have specific dates here for individuals and we don't really know to what degree this particular population may have been affected by these major events. While I do like the narrative overall, I think the authors should tread lightly on making this a make or break case of the impact of the LALIA. The linking of measles emergence to this event is also over drawn. The authors here are simply linking the tMRCA of modern measles with rinderpest. But we have no idea when along that branch measles actually emerged (line 241).

Response: Authors’ hypothesis concerning LALIA was toned down and removed from the Conclusion section. The link with measles emergence was removed from the manuscript.

Lines 316-317 on page 15 in the manuscript:

It is notable that LALIA coincides with several changes in the pathogen landscape. A case in point is the Justinian plague (541-543 AD)

Lines 323-326 on pages 15-16 in the manuscript:

Taken together, we would like to hypothesise that LALIA may have created an ecological context in which persistent outbreaks set the stage for severe epidemics hundreds of years later. This speculation, however, needs corroboration.

In line 340-342 on page 16 we have deleted the following sentence:

Thus, we hypothesize that LALIA may have been one of the factors that substantially contributed to the infectious disease epidemics which broke out in Europe several hundred years later.

12)The authors also point out the fact that the cementery abuts the Justinian plague, so it was surprising that no of the individuals had plague in them, is that true?

Response: It is correct, no Y. pestis DNA was found in any of the screened metagenomic samples. This information is now pointed out in the Discussion section:

Lines 278-280 on page 14 in the manuscript:

Although the dating of the Lauchheim settlement abuts the Justinian plague (541-543 AD), no traces of the causative Yersinia pestis (Y. pestis) have been discovered in any of the analysed samples. Moreover, in the case of the Lauchheim settlement, most likely not all inhabitants were buried within its borders and part of the population might have been buried in the traditional graveyard situated nearby or at another unknown location. Overall, the burials may not be representative of the general Lauchheim population.

13)The authors cite a reference (12) for all their methods, but they need to include those details or some of them here.

Response: the methods section was expanded and more details were added concerning the bioinformatic analysis.

Lines 360-368 on pages 17-18 in the manuscript (pathogen screening):

Subsequently, all generated metagenomic datasets were screened for the presence of pathogens with Megan Alignment Tool 0.3.0 (MALT) [28] (SemiGlobal alignment mode, identity threshold = 85%), using a custom database containing all bacterial and viral genomes from the NCBI website (download: 24.01.2019). Output alignments were inspected visually in MEGAN 6 [29]. Samples showing presence of pathogen DNA were further sequenced to obtain more data (Additional file 1: Tab. S3). Additionally, virus (111 human pathogens available on the NCBI website in 2018) targeted capture was performed on the VARV-positive sample (G31). Enriched data was then mapped against reference genomes of the respective pathogens to confirm the findings.

Lines 373-379 on page 18 in the manuscript (M. leprae analysis):

The pre-processed reads were mapped against the M. leprae TN reference genome (NC_002677) with the Burrows-Wheeler Aligner (BWA) v0.7.12 [30]. Output SAM files were converted to BAM format and merged, sorted and indexed with SAMtools v1.3 with default parameters [31]. Duplicated reads were removed using DeDup v0.12.2 [32] and the BAM file was filtered for quality 30. To generate VCF files, UnifiedGenotyper module from the Genome Analysis Toolkit (GATK) v3.6 was used. All the reads in the input were assigned to a single new read group with the Picard AddOrReplaceReadGroups tool with default parameters [33].

Lines 383-385 on page 18 in the manuscript (M. leprae analysis):

The phylogenetic M. leprae tree was produced with RaxML [34] with a bootstrap of 500 replicates (GTR- GAMMA model).

Lines 404-409 on page 19 in the manuscript (HBV analysis):

A dataset containing 109 representative modern HBV genomes, 17 ancient HBV genomes and the four genomes from Lauchheim was used for the generation of a phylogenetic tree. A multiple sequence alignment was made using MAFFT [35] and phylogenetic informative blocks were extracted using GBLOCKS with default parameters [36]. A maximum likelihood phylogeny was generated using PhyML with the GTR substitution model, estimated proportion of invariable sites, NNI tree topology search and 500 bootstrap replicates [37].

Lines 421-424 on page 20 in the manuscript (B19 analysis):

A MAFFT alignment followed by GBLOCKS was performed. A maximum likelihood phylogeny was generated using PhyML with the TN93 substitution model, estimated proportion of invariable sites, NNI tree topology search and 500 bootstrap replicates.

14)Finally the authors mention that there was no need for ethics approval for their destructive analyses, can they describe why that's the case in this situation?

Response: In Germany, an ethics approval is not required for work on archaeological human specimens. Our destructive analyses were limited to a minimum and performed with full approval from the curator and responsible archaeologists (Dirk Krausse, Jonathan Scheschkewitz) and anthropologists (Joachim Wahl, Michael Francken) of the Lauchheim material, all are co-authors of this study.

**2^nd^ round**

**Reviewer 1**

No further comments.

**Reviewer 2**

The authors have done an excellent job addressing the minutiae of my previous questions.

I have two very minor suggestions to this new draft. I look forward to seeing the paper in print.

1. Line 204 - the authors say they ID pathogens at the time of death. I don’t know if that’s necessarily true. To what degree does DNA of prior infections ‘hang’ out in teeth? While I agree it’s likely, I tend to lean on the side of caution. The authors can do as they see fit.

2. On lines 251-254 - the authors argue attempt to argue as to why they only see VARV in a single individual if it’s a communicable disease. They write “… lower fatality rate, or that viremia did not last during the infection enabling increased transmission…” . However given the fact that this strain is not epidemiologically smallpox, why couldn’t it simply be that this (like the other 4th-7thC) strains were simply not human-human communicable. That the evolutionary changes needed for that hadn’t yet happened and thus the infection rate remained low.

**Authors’ response**

Both comments from the Reviewer 2 were addressed within the manuscript. The title,

abstract, manuscript body and supplement were formatted according to the guidelines

of Genome Biology. The dataset analyzed in this study is cited in the reference list.
